# Efficacy and safety of acupuncture on sleep quality for post-stroke insomnia: a systematic review and meta-analysis

**DOI:** 10.3389/fneur.2023.1164604

**Published:** 2023-06-27

**Authors:** Menglong Shi, Zhaochen Ji, Tianye Sun, Haiyin Hu, Zhe Chen, Chaonan Feng, Junhua Zhang, Min Zhao, Fengwen Yang

**Affiliations:** ^1^Evidence-Based Medicine Center, Tianjin University of Traditional Chinese Medicine, Tianjin, China; ^2^Dongfang Hospital, Beijing University of Chinese Medicine, Beijing, China; ^3^Hospital of Brain Diseases, The First Affiliated Hospital of Henan University of CM, Zhengzhou, China

**Keywords:** acupuncture, post-stroke insomnia, sleep quality, meta-analysis, systematic review

## Abstract

**Background:**

Stroke is the second leading cause of death worldwide, and improving sleep quality in post-stroke insomnia is beneficial to the recovery of stroke. Acupuncture is widely used for the treatment of post-stroke insomnia in China. Therefore, this systematic review and meta-analysis were performed to explore the efficacy and safety of acupuncture for post-stroke insomnia.

**Methods:**

Eight databases were searched from their inception to 12 September 2022. Two reviewers independently performed the study screening and data extraction. The outcomes include Pittsburgh Sleep Quality Index (PSQI), objective sleep data measured by polysomnography (PSG), long-term efficacy and adverse events. The quality of the trials was assessed by the Cochrane risk of bias tool 2.0. The RevMan 5.4 and Stata 15.1 were used for data synthesis.

**Results:**

Among 3,233 participants from 41 studies were included. Pooled results indicated that acupuncture was superior to control group (CG) in improving PSQI total score (standardized mean difference (*SMD*) = −1.03, 95% confidence interval (CI): −1.32, −0.74, *P* < 0.00001), increasing sleep efficiency (*SMD* = 0.65, 95% CI: 0.37 to 0.92) and total sleep time (*SMD* = 0.54, 95% CI: 0.22 to 0.86). The favorable results in improving PSQI total score (*SMD* = 0.65, 95% CI: 0.37 to 0.92), reduced sleep latency (*SMD* = 1.84, 95% CI: 0.31 to 3.38) and increased total sleep time (*SMD* = −0.73, 95% CI: −1.15 to −0.31) were also observed in comparisons of acupuncture plus CG vs. CG. As of long-term efficacy and safety, the effects of acupuncture were long-term and robustness, however, due to limited safety information, reliable safety conclusions cannot be drawn. Subgroup analysis showed that acupuncture plus CG was superior to CG for post-infarction patients, but the efficacy of acupuncture alone compared to non-BZDs or other hypnotics needs further research. The GRADE assessment demonstrated that the level of evidence was mostly low or very low given the flaws in the study design and considerable heterogeneity among the included studies.

**Conclusion:**

Acupuncture could improve sleep quality, has long-term efficacy and without serious adverse events. However, the findings should be treated with caution owing to the existence of methodological quality issues. More studies with rigorous designs are warranted for validation and explored the safety of acupuncture.

## Graphical Abstract

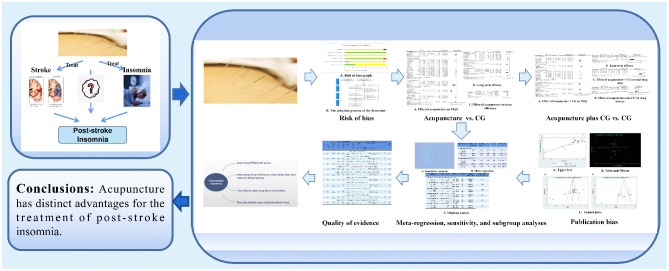

## 1. Introduction

Stroke is increasing in prevalence and is the second leading cause of death worldwide (1–3). Stroke patients are often left with lingering effects, including impairments in mobility, cognition, and communication (4, 5). Insomnia, the difficulty of falling and staying asleep, is the most common mental disorder and is highly prevalent in stroke patients (6). Changes in sleep are commonly reported following stroke, and the incidence of insomnia ranges from 38.2% to 40.7% in post-stroke patients (7). Several reviews have shown that sleep-wake disorders are both risk factors and consequences of stroke that modulate stroke recovery and outcomes, therefore, insomnia management is an essential component of stroke rehabilitation (8, 9). Timely treatment of post-stroke insomnia reduces the risk of cardiovascular events and improves motor and cognitive functions (10). However, it has barely received widespread attention, and research on the rehabilitation process is scarce.

The treatment of post-stroke insomnia patients is mainly based on lifestyle changes, including a quiet environment, moderate physical activity, and taking sleeping pills, if necessary (8). Benzodiazepines (BZDs), non-benzodiazepines (non-BZDs), and sedative antidepressants are commonly used sleeping pills; however, non-BZD and BZDs may worsen neuropsychological deficits and lead to the re-emergence of motor deficits (11). Several studies have shown that zolpidem use is associated with stroke risk (OR 1.50 for 470 mg zolpidem/y), and a high annual BZD dose (4 g) or long duration of BZD use (95 days) increased stroke incidence (12, 13). Cognitive behavioral therapy, the first choice of treatment for insomnia, has been shown to improve sleep quality in post-stroke insomnia in some cases; however, the results need to be treated with caution owing to the lack of large-sample studies (14). The use of cognitive behavioral therapy in the treatment of post-stroke insomnia has also been hindered by cognitive and mobility limitations. Owing to the limitations of current treatments, complementary and alternative medicines have been increasingly introduced into the treatment of post-stroke insomnia.

As a basic therapy used in traditional Chinese medicine for the prevention and treatment of diseases, acupuncture involves to inserting needles into acupuncture points or specific parts of the human body. Acupuncture theory holds that the human body is a whole connected by meridians, and that the physical stimulation of acupuncture points on these meridians can improve the body's ability to self-regulate, helping patients recover. Acupuncture can be used as a single intervention or in combination with other treatments, which is recommended for the treatment of stroke-related sequelae, such as hemiplegia (15), shoulder-hand syndrome (16), depression (17), post-stroke spasticity (18), and constipation (19). It is also widely used to treat primary and secondary insomnia (20, 21). While there is a high level of research-based evidence for acupuncture to treat insomnia or stroke-related sequelae, the existing systematic review (SR) conclusion on acupuncture to treat post-stroke insomnia still need to be applied with caution (22, 23). Studies have shown that acupuncture can improve the sleep quality of post-stroke insomnia patients. However, the methodological quality is low, included studies are incomplete, and intervention methods are not clear; therefore, there is a high risk of bias.

The present systematic review and meta-analysis comprehensively summarize the efficacy and safety of acupuncture as a monotherapy or as adjunctive therapy in the treatment of post-stroke insomnia to guide evidence-based clinical decision-making.

## 2. Materials and methods

This study has been registered in PROSPERO (number CRD42022364479) and was conducted accordance to the Preferred Reporting Items for Systematic Reviews and Meta-analysis (PRISMA) statement (24). The method is available from https://www.crd.york.ac.uk/prospero/.

### 2.1. Search strategy

Four English databases (PubMed, Embase, CENTRAL, and Web of Science) and four Chinese databases (SinoMed, VIP information database, China National Knowledge Infrastructure, and Wanfang Data Information Site) were searched independently by two independent researchers (Shi ML and Sun TY). The key words used included the following combination of medical subject headings (MeSH) and free text terms: “acupuncture therapy,” “acupuncture,” “electro-acupuncture,” “insomnia,” “sleep disturbance,” “stroke,” “cerebral infarction” and “randomized controlled trial” as the main subject headings or text words in titles and abstracts. The search time was established as 12 September 2022 (The strategy for all databases is described in Supplementary material). Furthermore, we searched the clinical trial registries such as the ClinicalTrials.gov registry and the Chinese Clinical Trial Registry.

### 2.2. Inclusion and exclusion criteria

#### 2.2.1. Types of studies

All randomized control trials (RCTs) published in English or Chinese were included. Others studies such as case reports and animal studies were excluded.

#### 2.2.2. Types of participants

Participants who were post-stroke and met the diagnostic criteria for insomnia were included. (1) Stroke was diagnosed according to the acknowledged criteria. (2) Patients met the diagnostic criteria for insomnia, such as the *Diagnostic and Statistical Manual of Mental Disorders-fifth edition (DSM-5), International Classification of Sleep Disorders (ICSD)*, and *International Statistical Classification of Diseases and Health Related Problems-tenth edition (ICD-10)*. (3) There were no restrictions on age, gender, race, or stroke type. However, primary insomnia or secondary insomnia due to the other causes were excluded.

#### 2.2.3. Types of interventions

The experimental group received acupuncture as a monotherapy or adjunctive therapy, which included manual acupuncture, electroacupuncture, body needle, and ear acupuncture. However, non-invasive methods such as cupping therapy were excluded.

#### 2.2.4. Types of comparisons

Patients in control group (CG) were treated with sham acupuncture or western medicine. However, other traditional medicine medicine therapy such as Chinese herb, massage, scraping, cupping were excluded.

#### 2.2.5. Outcome measures

The primary outcome was the Pittsburgh sleep quality index (PSQI) score, which has good internal consistency and test-retest reliability for insomnia patients (25). Furthermore, we collected the objective sleep data which was measured by polysomnography (PSG), such as sleep onset latency, sleep efficiency and total sleep time. Using data from follow-up visits and adverse events, we evaluated the long-term effects and safety of the acupuncture. The included studies had at least one of these outcomes.

### 2.3. Literature screening and data extraction

NoteExpress was used to manage the retrieved records and removed duplicates. Two researchers (Shi ML and Sun TY) independently performed the study screening and data extraction according to the criteria. Firstly, they eliminate irrelevant records based on the topics or abstracts, and then read the full-text to identify the eligible studies.

The data extracted were as follows: (1) Sample characteristics: authors, publication year, sample size, mean age, type of stroke, insomnia duration, allocation, and control conditions. (2) Study design: randomization, allocation concealment and blinding. (3) Acupuncture therapy: frequency, course, acupuncture points and retention time of acupuncture. (4) Information of outcomes: PSQI, sleep efficiency, sleep latency, total sleep time, follow-up visits and adverse reaction. The third researcher (Ji ZC) reconfirmed the final data extraction sheets. For missing data, we attempted to contact the authors of the article; otherwise, the study had to be excluded.

### 2.4. Quality of acupuncture treatment regimen in included trials

Two researchers (MS and TS) assessed the acupuncture treatment regimen in each study using the Standards for Reporting Interventions in Clinical Trials of Acupuncture (STRICTA) (26), in which six domains were evaluated: (1) acupuncture rationale, (2) details of needling, (3) treatment regimen, and other components of treatment such as (4) compactor intervention, (5) practitioner background, and (6) control intervention. Furthermore, we calculated frequency statistics on the acupuncture points included in the literature.

### 2.5. Risk of bias assessment

Two researchers (MS and TS) assessed the quality of each included study using the Cochrane risk of bias tool 2.0 (RoB2.0), in which six domains were evaluated: (1) randomization process; (2) deviations from the intended interventions; (3) missing outcome data; (4) measurement of the outcome; (5) selection of the reported outcome; (6) overall bias. Two reviewers (MS and TS) resolved discrepancies by discussion, and when necessary, discussion with the third researcher (ZJ), each entry was either rated as low risk, high risk, or some risk.

### 2.6. Data synthesis and analysis

Data conversion was performed in accordance with the Cochrane Handbook for Systematic Reviews of Interventions. If the study reported data at baseline and post-intervention, we calculated the group mean and standard deviation for each sleep measure. Moreover, when studies reported multiple arms of two kinds of acupuncture and control methods (e.g., special acupuncture [Xingnao Kaiqiao acupuncture], ordinary acupuncture, and CG), we combined the data from the two acupuncture groups. Finally, in cases where the studies reported time in hours, we converted it into minutes.

Statistical analysis were performed using Review Manager version 5.4 and Stata Statistical Software: Release 15.1 (StataCorp LLC., College Station, Texas). Heterogeneity between studies was quantified using the inconsistency index (*I*^2^), with an *I*^2^ value of >50% representing substantial heterogeneity. When *I*^2^ < 50%, *P* > 0.1, we used fixed-effect model to pool data; otherwise, the random-effect model was used. We calculated the standardized mean difference (SMD) between the acupuncture and control groups. Confidence intervals (CIs) were set at 95%, and a *p*-value < 0.05 indicated statistical significance for an overall effect.

### 2.7. Subgroup analysis

To determine whether certain characteristics influence the efficacy of acupuncture treatment, we planned to conduct subgroup meta-analysis. The main characteristics included: (1) the types of experimental intervention (acupuncture vs. CG, acupuncture plus CG vs. CG); (2) the types of CG (BZDs, non-BZDs, Selective Serotonin Reuptake Inhibitor (SSRIs), sham acupuncture); (3) the points of follow-up time (1st month after treatment, 2nd month after treatment, 2rd month after treatment). In addition, we performed subgroup analysis based on others characteristics to explore potential sources of heterogeneity, such as: (1) total sessions of treatment (course < 4 weeks, course ≥4 weeks); (2) acupuncture frequency (< 1 time/day, 1 time/day); (3) needle retention time (time < 30 min, time ≥30 min).

### 2.8. Sensitivity and meta-regression analysis

We performed sensitivity analysis by removing study one by one to identify the robustness of the result. In addition, we performed a meta-regression using sample size, mean age, and year of publication as co-variables to query the sources of heterogeneity.

### 2.9. Publication bias

If the number of studies was >10 for comparison, we assessed the possibility of publication bias using funnel plots and assessed funnel plot asymmetry using Egger's regression test (*P* < 0.1). In addition, we performed trim-and-fill analysis to assess the effect of publication bias on the interpretation of the results.

### 2.10. Quality of evidence

We used the Grading of Recommendations Assessment, Development, and Evaluation (GRADE) approach to assess the certainty of the evidence for each outcome, in which five domains were evaluated: (1) study limitations were assessed according to the RoB2.0; (2) consistency was evaluated using the *I*^2^ values and the agreement of 95% confidence and prediction intervals; (3) directness was assessed to determine whether the interventions and populations of the included studies were appropriate for the research question; (4) precision was examined by the optimal information sample size (OIS) and by determining whether clinical decisions may differ if the true effect is at the upper or lower end of the 95% CI; (5) publication bias was assessed using the funnel plot and the number of included studies (27).

## 3. Results

### 3.1. Results of study selection

**Figure 1:**
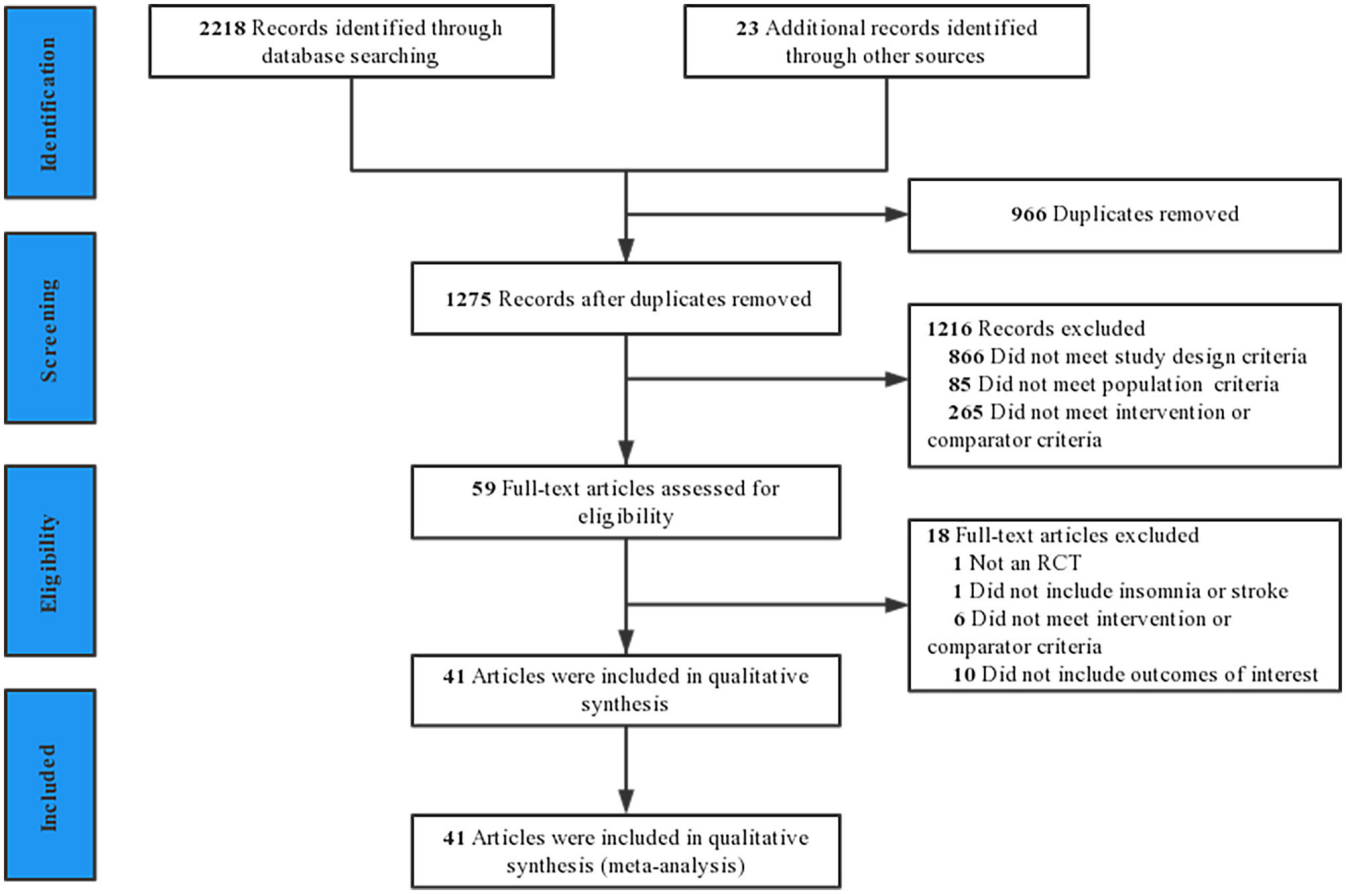
The selection process of the literature.

The results of the literature search and screening process are shown in Figure 1. The initial search 2,241 potential titles. Among them, 966 were duplicates and 1,275 were excluded after reading the title or abstract. In total, 59 clinical trials were reviewed at the full-text level for further evaluation, of which 18 were excluded. Finally, 41 studies were included in the final review (28–68). The excluded 18 studies and reasons for exclusion are provided in Supplementary Table S9.

### 3.2. Characteristics of the included studies

A total of 41 studies involving 3,262 patients were identified (Table 1), however, 29 participants dropped from five studies (31, 32, 36, 45, 50). Finally, data from 1,742 patients in the intervention group and 1,491 patients in the control group were included in the meta-analysis. All included RCTs were conducted from 2004 to 2022. The mean age of the participants was 42–75 years. As for stroke type, ten trials (29–31, 34, 35, 38, 39, 45, 49, 51, 52) recruited patients with cerebral ischemia and five (46, 50, 53, 58, 63, 67) studies clarified the number of patients with cerebral ischemia and cerebral hemorrhage in each group. The other studies did not report the type of stroke.

**Table 1:** Detailed information about the studies included.

**Study (Year)**	**Participants' characteristics (baseline)**		**Intervention**		**Main results**	**Adverse events**
	**Acupuncture**	**Control**	**Acupuncture**	**Control**		
Mai et al. (28)	N = 31 M/F = 14/17 A = 60.22 ± 9.37 ST = NR	N = 31 M/F = 15/16 A = 59.45 ± 8.36 ST = NR	Type = MA+CON Duration = 4 w F = 1 time/2 d NRT = 30 min	ESZL	PSQI	
Chen et al. (29)	N = 49 M/F = 26/23 A = 60.78 ± 5.24 ST = CI	N = 49 M/F = 28/21 A = 61.03 ± 5.01 ST = CI	Type = MA+CON Duration = 4 w F = 1 time/d NRT = 30 min	ESZL	PSQI, TST	
Zhang (35)	N = 33 M/F = 21/12 A = 54.35 ± 9.04 ST = CI	N = 33 M/F = 19/14 A = 54.25 ± 8.89 ST = CI	Type = MA+CON Duration = 2 w F = NR NRT = 30 min	ESZL	PSQI TST	
Zhan et al. (31)	N = 44 M/F = 17/27 A = 65.22 ± 9.94 ST = CI	N = 43 M/F = 25/18 A = 65.47 ± 9.31 ST = CI	Type = EA+CON Duration = 4 w F = 1 time/d NRT = 30 min	EZPCL	PSQI	
Yao et al. (33)	N = 30 M/F = 23/7 A = 59.32 ± 9.27 ST = NR	N = 30 M/F = 20/10 A = 61.40 ± 9.04 ST = NR	Type = ME+CON Duration = 4 w F = 1 time/2 d NRT = 24 h	EZPCL	PSQI	
Li (32)	N = 36 M/F = 18/16 A = 69.41 ± 4.58 ST = NR	N = 36 M/F = 23/13 A = 68.28 ± 5.04 ST = NR	Type = MA+CON Duration = 2 w F = 1 time/d NRT = 30 min	EZPCL	PSQI	
Zhang et al. (46)	N = 39 M/F = 23/16 A = 52.48 ± 4.13 ST = NR	N = 39 M/F = 21/18 A = 51.63 ± 3.54 ST = NR	Type = MA+CON Duration = 4 w F = 1 time/d NRT = 30 min	APZL	PSQI	
Wu et al. (37)	N = 38 M/F = 16/22 A = 58.22 ± 3.42 ST = NR	N = 38 M/F = 15/23 A = 59.31 ± 3.51 ST = NR	Type = MA+CON Duration = 4 w F = 1 time/d NRT = 30 min	ESZL	PSQI	
Wang (44)	N = 30 M/F = 19/11 A = 62.83 ± 11.26 ST = NR	N = 30 M/F = 17/13 A = 63.33 ± 10.75 ST = NR	Type = MA+CON Duration = 4 w F = 1 time/d NRT = 20–30 min	DZP	PSQI	
Sun et al. (39)	N = 44 M/F = 31/13 A = 56.17 ± 6.25 ST = CI	N = 44 M/F = 28/16 A = 56.39 ± 6.09 ST = CI	Type = MA+CON Duration = 8w F = NR NRT = 30 min	No	PSQI	
Hu et al. (50)	N = 58 M/F = 36/22 A = 41-69 ST = CI47 CH11	N = 59 M/F = 35/24 A = 43-68 ST = CI48 CH13	Type = MA+CON Duration = 3w F = NR NRT = 30-40min	ESZL	PSQI	
Wang et al. (52)	N = 76 M/F = 40/36 A = 64 ± 6 ST = CI	N = 76 M/F = 37/39 A = 64 ± 6 ST = CI	Type = MA+CON Duration = 4 w F = 1 time/d NRT = 20–30 min	EZPCL	PSQI TST SE	
Liu et al. (53)	N = 36 M/F = 20/16 A = 52.31 ± 8.2ST = CI22 CH14	N = 36 M/F = 24/12 A = 52.08 ± 6.1 ST = CI19 CH17	Type = MA+CON Duration = 4 w F = 1 time/3d NRT = NR	ESZL	PSQI	
Han (58)	N = 28 M/F = 14/14 A = 60.75 ± 6.87 ST = CI23 CH5	CON1 N = 28 M/F = 15/13 A = 62 ± 6.72 ST = CI20 CH8 CON2 N = 28 M/F = 17/12 A = 63.69 ± 5.1 ST = CI22 CH7	Type = MA+CON1 Duration = 4 w F = 1 time/d NRT = 30 min	**CON1** = ESZL **CON**2 = MA+**CON1**	PSQI	0 in ACU 0 in CON
Liu et al. (30)	N = 33 M/F = 16/17 A = 65.44 ± 7.80 ST = NR	N = 33 M/F = 18/15 A = 65.16 ± 7.84 ST = NR	Type = MA+CON Duration = 4 w F = 1 time/d NRT = 30 min	ESZL	PSQI	
Xing et al. (34)	N = 40 M/F = 24/16 A = 63.25 ± 9.74 ST = NR	N = 40 M/F = 21/19 A = 62.14 ± 8.59 ST = NR	Type = MA Duration = 4 w F = 1 time/d NRT = 25 min	ESZL	PSQI	
Zou et al. (41)	N = 30 M/F = 16/14 A = 57.20 ± 12.72 ST = NR	N = 30 M/F = 17/13 A = 61.23 ± 10.85 ST = NR	Type = MA Duration = 3 w F = NR NRT = NR	ESZL	PSQI	
Wang et al. (43)	N = 32 M/F = 15/17 A = 66 ± 8 ST = NR	CON1 N = 30 M/F = 15/15 A = 67 ± 8 ST = NR CON2 N = 30 M/F = 14/16 A = 65 ± 9 ST = NR	Type = MA Duration = 4 w F = 1 time/d NRT = 30 min	**CON1** **=** ESZL **CON2** **=** MA	PSQI	
Sun (42)	N = 30 M/F = 16/14 A = 63.07 ± 7.89 ST = NR	N = 30 M/F = 13/17 A = 60.13 ± 7.17 ST = NR	Type = MA Duration = 4 w F = 1 time/d NRT = NR	EZPCL	PSQI	2 in ACU: acupuncture syncope, hematoma 1 in CON:hematoma
Ye et al.(36)	N = 37 M/F = 9/28 A = 58.7 ± 8.11 ST = NR	CON1N = 36 M/F = 11/25 A = 58.53 ± 6.23 ST = NR CON2N = 38 M/F = 12/26 A = 58.37 ± 6.97 ST = NR	Type = MA Duration = 4 w F = 1 time/d NRT = 30 min	**CON1** **=** ESZL **CON2** **=** MA	PSQI	6 in ACU, 6 in CON1N, 13 in CON2N (minor adverse reactions occurred, and no treatment was needed)
He et al.(40)	N = 30 M/F = NR A = NR ST = NR	CON1 N = 30 M/F = NR A = NR ST = NR CON2 N = 30 M/F = NR A = NR ST = NR	Type = MA Duration = 2w F = 1 time/d NRT = 30–40 min	**CON1** **=** ESZL **CON2** **=** MA+CON1	PSQI	
Cao et al. (38)	N = 6 M/F = 23/13 A = 63 ± 9 ST = NR	N = 36 M/F = 30/6 A = 63 ± 6 ST = NR	Type = MA Duration = 4 w F = 1 time/d NRT = 30 min	SA	PSQI TST SE	
Cao et al. (45)	N = 43 M/F = 27/16 A = 68.4 ± 5.1 ST = NR	N = 41 M/F = 25/16 A = 67.3 ± 5.9 ST = NR	Type = MA Duration = 4 w F = 1 time/d NRT = 30 min	SA	PSQI TST SE	4 in ACU: bleeding 0 in CON
Yuan (48)	N = 30 M/F = 16/14 A = 61.86 ± 6.44 ST = NR	N = 30 M/F = 17/13 A = 61.83 ± 5.64 ST = NR	Type = MA Duration = 3w F = 1 time/d NRT = 20 min	ESZL	SE	
Lai (49)	N = 28 M/F = 15/13 A = 53.21 ± 7.48 ST = NR	N = 28 M/F = 17/11 A = 55.68 ± 8.45 ST = NR	Type = MA Duration = 2w F = 1 time/d NRT = 30 min	ESZL	PSQI	
Huang et al. (47)	N = 30 M/F = 17/13 A = 60.17 ± 10.12 ST = NR	N = 30 M/F = 16/14 A = 59.47 ± 8.34 ST = NR	Type = MA Duration = 4 w F = 1 time/d NRT = 30–40 min	ESZL	PSQI	
Yang et al. (51)	N = 30 M/F = 18/12 A = 70.09 ± 12.9 ST = NNR	N = 30 M/F = 22/8 A = 67.50 ± 11.57 ST = NR	Type = MA Duration = 2w F = 1 time/d NRT = NR	ESZL	PSQI	
Ma et al. (54)	N = 40 M/F = 24/16 A = 61.88 ± 5.16 ST = NR	CON1N = 40 M/F = 20/20 A = 63.70 ± 4.94 ST = NR CON2N = 40 M/F = 18/22 A = 63.33 ± 4.43 ST = NR	Type = MA Duration = 4 w F = 1 time/d NRT = 30 min	**CON1** **=** ESZL **CON2** **=** MA	PSQI	6 in ACU: dizziness, lethargy, constipation, perspiration 6 in CON1N: nausea, dizziness, lethargy, constipation, perspiration 29 in CON2N: nausea, dizziness, lethargy, constipation, gait instability, perspiration, thirst
Fu et al. (55)	N = 40 M/F = 23/17 A = 61 ± 9 ST = NR	N = 40 M/F = 22/8 A = 67.50 ± 11.57 ST = NR	Type = MA Duration = 4 w F = 1 time/d NRT = 20 min	PX	PSQI	
Tang et al. (57)	N = 34 M/F = 18/16 A = 58.25 ± 9.31 ST = NR	N = 31 M/F = 17/14 A = 59.68 ± 8.73 ST = NR	Type = MA Duration = 4 w F = 1 time/d NRT = 30 min	ESZL	PSQI	
Gou et al. (56)	N = 30 M/F = NR A = NR ST = NR	CON1 N = 30M/F = NR A = NR ST = NR CON2 N = 30 M/F = NR A = NR ST = NR	Type = MA Duration = 4 w F = NR NRT = NR	**CON1** **=** ZPCL **CON2** **=** MA	PSQI	
Zhu (60)	N = 30 M/F = 30/22 A = 75.2 ± 1.3 ST = NR	N = 44 M/F = 26/18 A = 74.3 ± 2.9 ST = NR	Type = MA Duration = 4 w F = 1 time/d NRT = 20-30 min	DZP	PSQI	
Jiang (59)	N = 49 M/F = 20/29 A = 63.88 ± 8.06 ST = NR	N = 50 M/F = 23/27 A = 61.06 ± 8.71 ST = NR	Type = MA Duration = 4 w F = NR NRT = 30 min	ESZL	PSQI	
Xu YL et al. (62)	N = 30 M/F = 16/14 A = 62.48 ± 3.66 ST = NR	N = 30 M/F = 17/13 A = 62.4 ± 6.78 ST = NR	Type = MA Duration = 4 w F = 1 time/d NRT = 30 min	DZP	PSQI	
Chen et al. (61)	N = 35 M/F = 18/17 A = 70 ± 8 ST = NR	CON1 N = 35M/F = 20/15 A = 70 ± 8 ST = NR CON2 N = 35M/F = 21/14 A = 69 ± 7 ST = NR	Type = MA Duration = 4 w F = 1 time/d NRT = 20min	**CON1** = ESZL **CON2** = MA	PSQI	
Wang (63)	N = 40 M/F = 21/19 A = 61.55 ± 2.1 ST = NR	N = 38 M/F = 17/21 A = 63.15 ± 2.5 ST = NR	Type = MA Duration = 4 w F = 1 time/d NRT = 30 min	ESZL	PSQI	
Jia (2010) (64)	N = NR M/F = NR A = NR ST = NR	N = NR M/F = NR A = NR ST = NR	Type = MA Duration = 4 w F = 1 time/d NRT = 30 min	ESZL	PSQI	0 in ACU 3 in CON: thirst, dependence
Mi et al.(65)	N = 40 M/F = 27/13 A = 65.28 ± 10.54 ST = NR	N = 40 M/F = 24/16 A = 63.11 ± 11.96 ST = NR	Type = MA Duration = 4 w F = 1 time/d NRT = 30 min	ESZL	PSQI	
Lu et al.(66)	N = 25 M/F = 14/11 A = 61.48 ± 3.72 ST = NR	N = 25 M/F = 15/10 A = 62.40 ± 4.88 ST = NR	Type = MA Duration = 4 w F = 1 time/d NRT = 30 min	DZP	PSQI	
Li (67)	N = 32 M/F = 18/14 A = 69.8 ± 7.1 ST = NR	N = 32 M/F = 17/15 A = 67.3 ± 8.3 ST = NR	Type = MA Duration = 4 w F = 1 time/d NRT = 20–30 min	ESZL	PSQI	
Wang et al. (68)	N = 34 M/F = 22/12 A = 42.5–70.5 ST = NR	N = 30 M/F = 17/13 A = 41-70 ST = NR	Type = MA Duration = 4 w F = 1 time/d NRT = 20–30 min	DZP	PSQI	

A, age; ACU, acupuncture group; APZL, alprazolam; CON, control group; CH, intracerebral hemorrhage; CI, cerebral ischemia; DZP, diazepam; EA, electroacupuncture; ESZL, Estazolam; EZPCL, Eszopiclone; F, frequency; MA, manual acupuncture; M/F, male/female; N, number of participants; NR, not reported; NRT, needle retention time; PSG, polysomnography; PSQI, Pittsburgh sleep quality index; PX, paroxetine; SA, sham acupuncture; SE, sleep efficiency; ST, stroke type; TST, total sleep time.

In all included studies, seven studies (36, 40, 43, 54, 56, 58, 61) consisted of three arms, five (36, 54, 56, 58, 61) of which involved the comparisons of special acupuncture (Xingnao Kaiqiao acupuncture) vs. ordinary acupuncture vs. CG. One study (40) involved the comparisons of acupuncture plus moxibustion vs. acupuncture vs. CG. One study (43) involved the comparisons of acupuncture plus CG vs. acupuncture vs. CG. In control groups, western medicine such as long-acting BZDs (diazepam), medium-acting BZDs (estazolam), short-acting BZDs (alprazolam), non-BZDs (zopiclone), SSRI (paroxetine) were used as interventions; Furthermore, two studies (38, 45) used sham acupuncture (retractable non-penetrating needles) as control intervention.

### 3.3. Acupuncture treatment regimen in included trials

A total of 54 acupuncture points were used in included studies. The most commonly used acupuncture points for post-stroke insomnia were GV20, HT7, SP6, GV24, EX-HN1, PC6, Anshen point, and GV29 (The sequencing of acupuncture points are provided in Supplementary Table S10). We used the STRICTA criteria to evaluate acupuncture treatment protocols (Supplementary Table S11); the results showed that all studies reported acupuncture rationale, treatment regimens, and control interventions. However, the practitioner's background, setting and context of treatment, the details of needling, such as the number of needles inserted, the selection of unilateral and bilateral points, responses elicited, and needle type were not detailed.

### 3.4. Risk of bias

**Figure 2:**
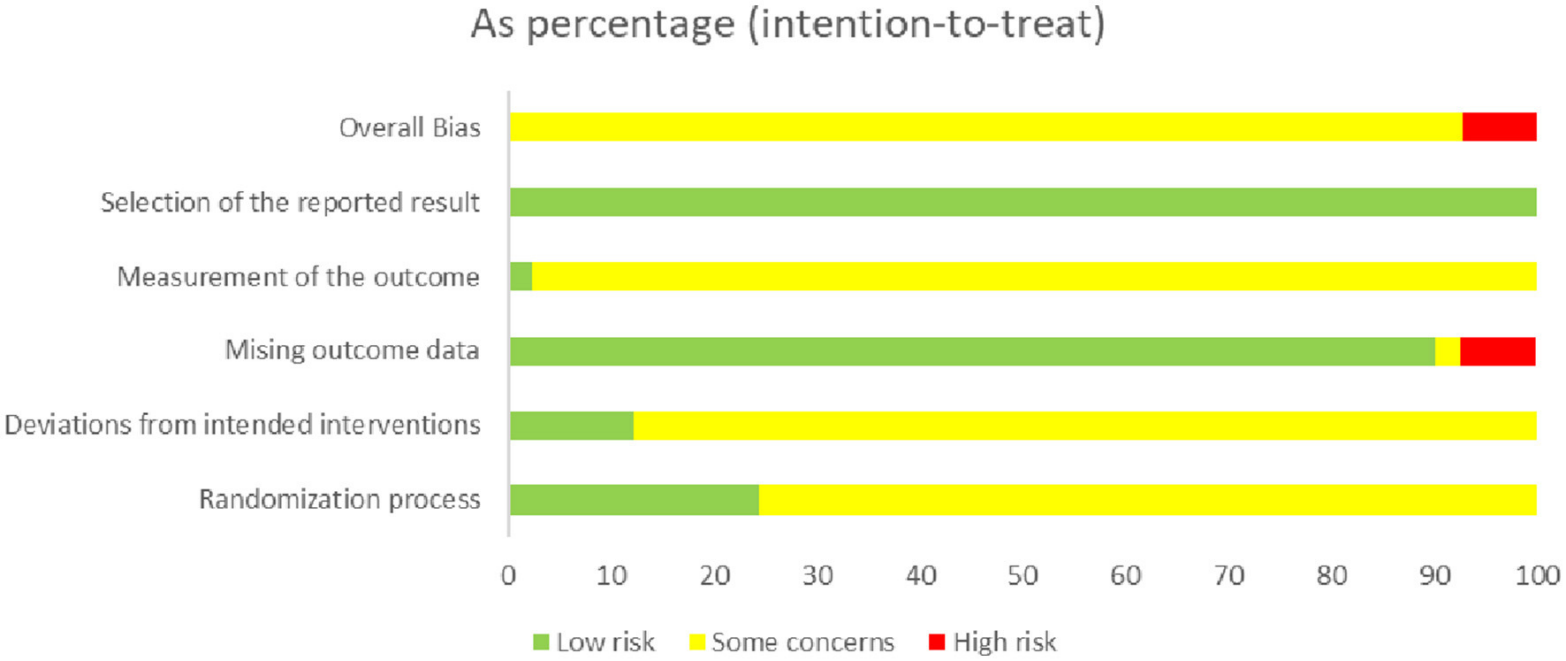
Risk of bias.

Three studies (32, 36, 45) had a domain rated as having an extreme risk of bias. All studies had at least one domain rated as having an uncertain risk of bias due to a lack of information. An adequate randomization process was described in all studies. In addition, ten studies (32, 38, 49, 52, 55, 58, 59, 61, 67, 68) used sealed envelopes or central randomization for allocation concealment. We only assessed participant blinding owing to the difficulty of blinded therapists in acupuncture therapy. Two studies (38, 45) used a participant-blinded design (from real and sham acupuncture groups). Five studies (31, 32, 36, 45, 50) described withdrawals and dropouts; one of the studies (50) did not report the cause of dropout and was rated as having an unknown risk. Patients in one study (32) dropped out of the trial owing to poor efficacy, and two studies had a high number of dropouts (36, 45), which could lead to higher risks. None of the studies provided information on selective reporting. The risk of bias is summarized in Figure 2.

### 3.5. Primary outcomes

#### 3.5.1. Acupuncture vs. CG

**Figure 3:**
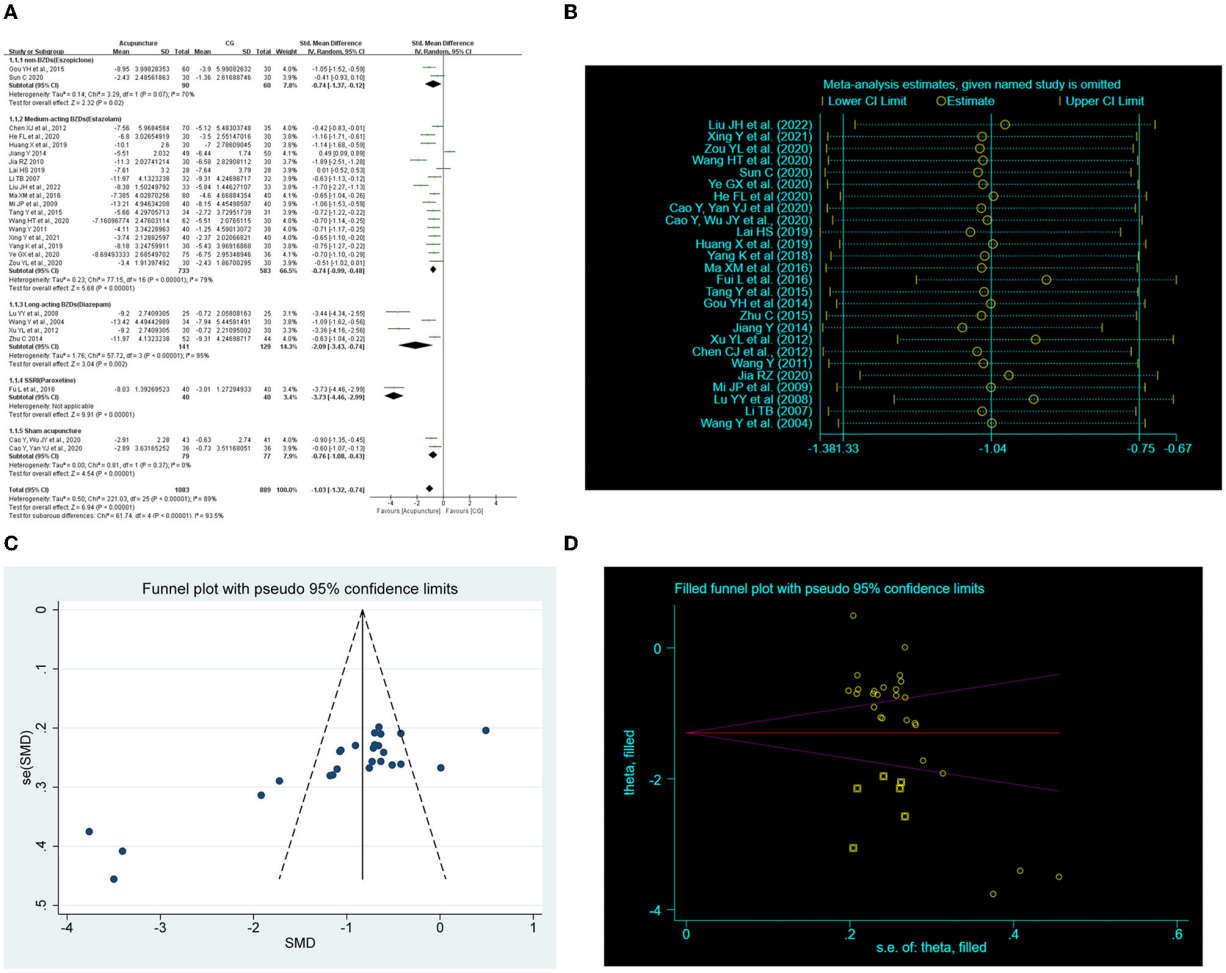
Effect of acupuncture on total Pittsburgh Sleep Quality Index (PSQI) score. **(A)** Forest plot of total PSQI score. **(B)** Sensitivity analysis of total PSQI score. **(C)** Funnel plot analysis revealed potential publication bias. **(D)** Trim-and-fill test analysis to assess the effect of publication bias on the interpretation of the results.

Twenty-six RCTs (19, 30, 34, 36, 38, 40–43, 45, 47, 49, 51, 54–57, 59–61, 63–68) examined the improvement in total PSQI score for acupuncture compared with CG. The random-effects model was used because of the notable heterogeneity. The meta-analysis showed markedly improvement of acupuncture on the PSQI total score (*SMD* = −1.03, 95% CI: −1.32 to −0.74, *P* < 0.00001, Figure 3A). Furthermore, we conducted subgroup meta-analysis to determine whether the types of control interventions influence the efficacy of acupuncture treatment. The result showed marked improvement with acupuncture on the total PSQI score compared with those of non-BZDs (eszopiclone), medium-acting BZDs (estazolam), long-acting BZDs (diazepam), and sham acupuncture. One study (55) showed that acupuncture had a better total PSQI score improvement than an SSRI (paroxetine). Sensitivity analysis was also carried out by excluding studies one by one, and the results showed the meta-analysis results were not altered, suggesting that the results were robustness (Figure 3B).

### 3.6. Acupuncture plus CG vs. CG

**Figure 4:**
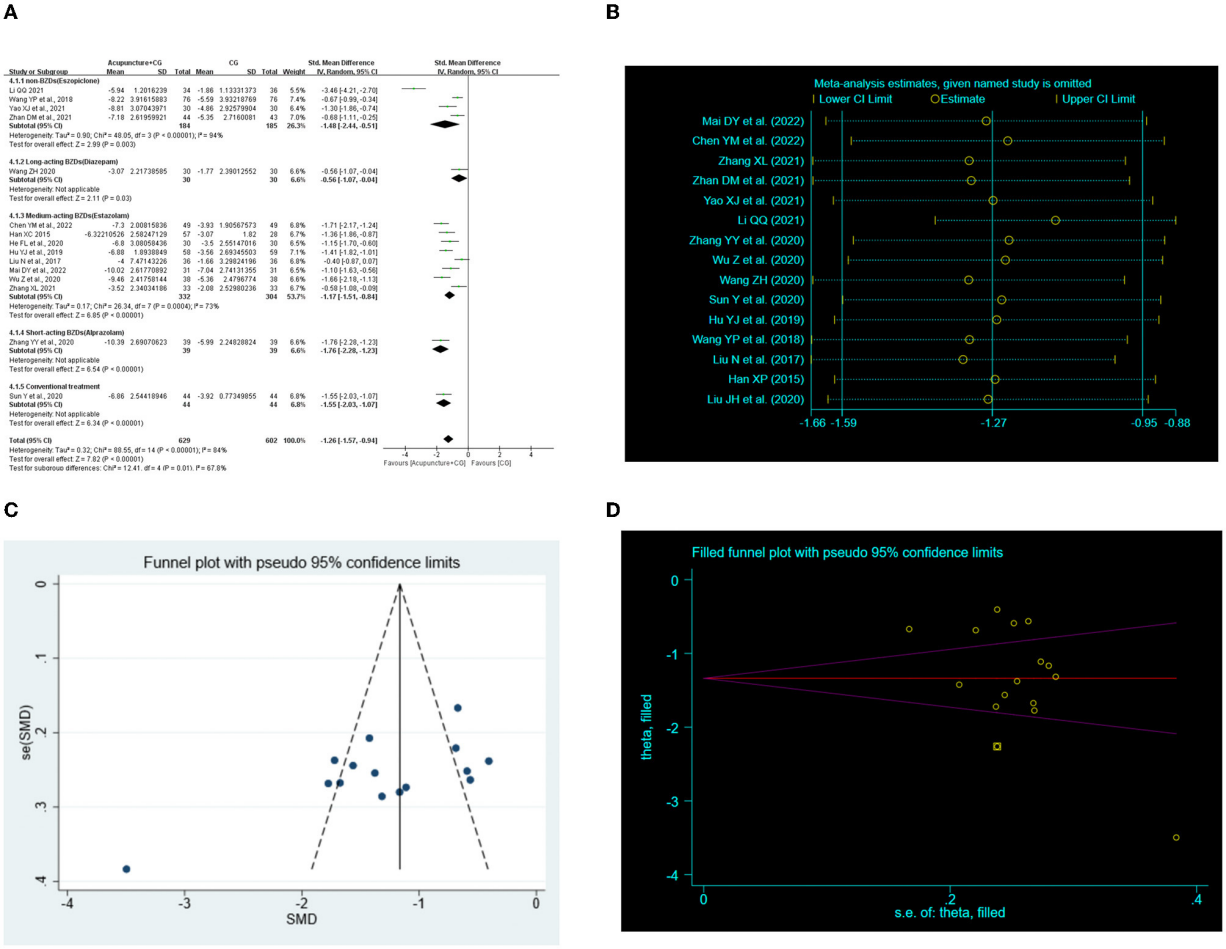
Effect of acupuncture plus control group (CG) on total Pittsburgh Sleep Quality Index (PSQI) score. **(A)** Forest plot of total PSQI score. **(B)** Sensitivity analysis of total PSQI score. **(C)** Funnel plot analysis revealed potential publication bias. **(D)** Trim-and-fill test analysis to assess the effect of publication bias on the interpretation of the results.

Fifteen RCTs (28, 29, 31–33, 35, 37, 39, 40, 44, 46, 50, 52, 53, 58) examined the improvement in the total PSQI score for acupuncture plus CG compared with CG. The random-effects meta-analysis showed a marked improvement of acupuncture plus CG on the total PSQI score (*SMD* = −1.26, 95% CI: −1.57 to −0.94, *P* < 0.00001, Figure 4A). Furthermore, we conducted subgroup analysis according to the intervention of control groups, and the results showed superior effects of acupuncture plus CG on the total PSQI score compared with non-BZDs (eszopiclone) and medium-acting BZDs (estazolam). Two studies (44, 46) showed that acupuncture plus long-acting BZDs (diazepam) and acupuncture plus short-acting BZDs (alprazolam) had a better total PSQI score improvement than the control group. Sensitivity analysis was also carried out, and the results suggested that the results were robustness (Figure 4B).

### 3.7. Subgroup analysis

**Figure 5:**
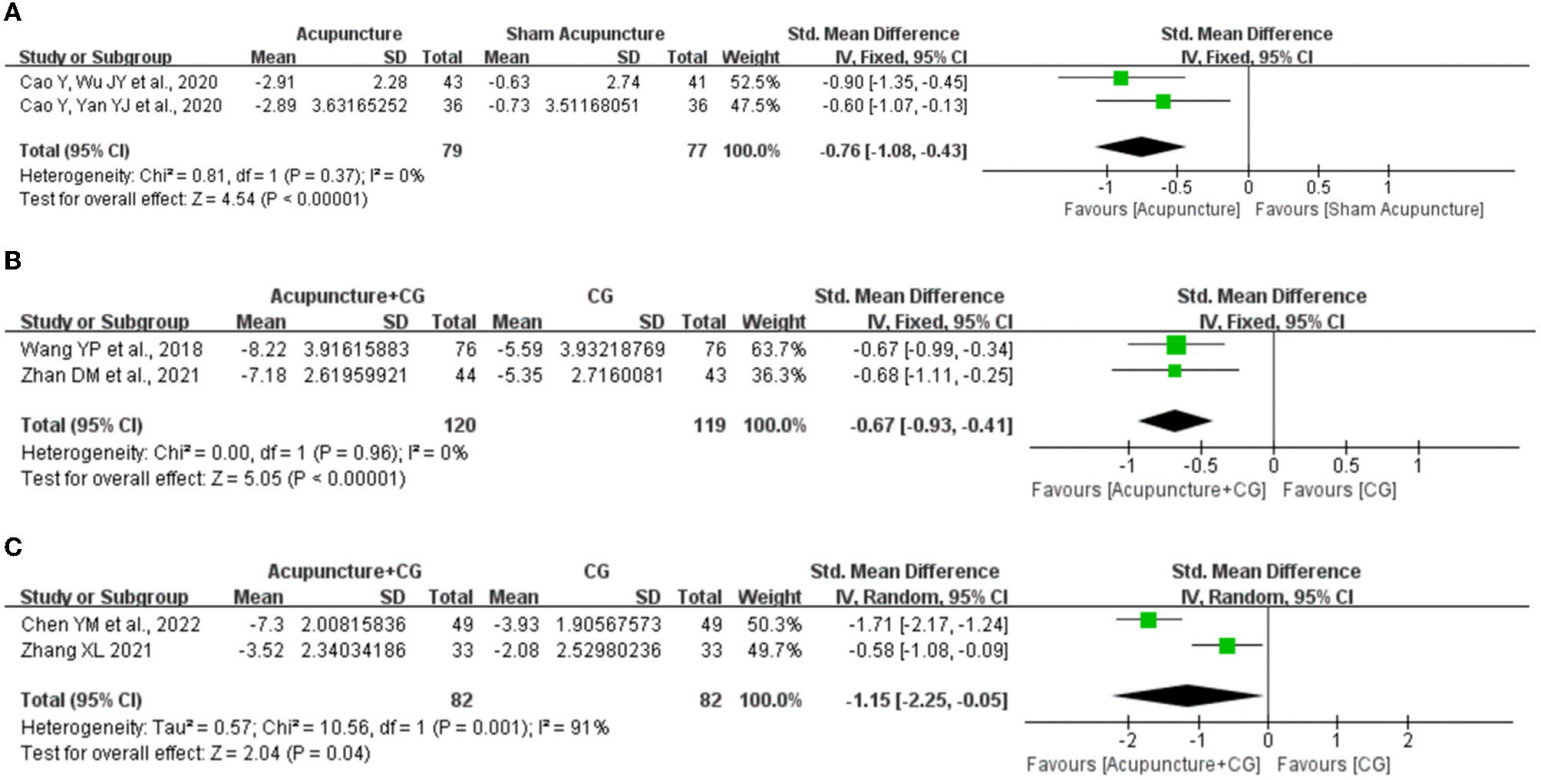
Effect of acupuncture for post-stroke insomnia. **(A)** Acupuncture vs. sham acupuncture. **(B)** Acupuncture plus non-BZDs vs. non-BZDs. **(C)** Acupuncture plus medium-acting BZDs vs. medium-acting BZDs.

Post-infarction patients were the most represented subgroup of post-stroke patients, we evaluated the effects of acupuncture on post-infarction insomnia. Five studies (30, 34, 38, 45, 49) evaluated the improvement in total PSQI score of acupuncture for post-infarction insomnia, and the results showed marked improvement of acupuncture compared with sham acupuncture (*SMD* = −0.76, 95% CI: −1.08 to −0.43, *P* < 0.00001), when we attempted to pool the results of acupuncture compared with medium-acting BZDs (estazolam), the sensitivity analysis found that one study (49) significantly affected the stability of the results, therefore, we conducted descriptive analysis and the result showed that two RCTs reported (30, 34) superior effects of acupuncture, and the other one (49) reported equivalent effects. Furthermore, five studies (29, 31, 35, 39, 52) evaluated the improvement in PSQI total score of acupuncture plus CG for post-infarction insomnia, and the result indicated acupuncture plus non-BZDs (eszopiclone) (*SMD* = −0.67, 95% CI: −0.93 to −0.41, *P* < 0.0001) or plus medium-acting BZDs (estazolam) (*SMD* = −1.15, 95% CI −2.25 to −0.05, *P* = 0.04) had a better PSQI total score improvement than the control intervention (Figure 5). Furthermore, subgroup analysis of the two interventions for PSQI total scores was also conducted based on the total sessions of treatment (course < 4 weeks, course ≥ 4 weeks), acupuncture frequency (< 1 time/day, 1 time/day), and needle retention time (time < 30 min, time ≥30 min). The results showed that the analysis of each subgroup was consistent with the overall results. However, as for acupuncture plus CG vs. CG, the difference in interactive effect was significant in PSQI with different needle retention time (Table 2).

**Table 2:** Subgroup analysis of the PSQI total scores for the two interventions.

**Subgroup**	***N***	**SMD**	**95% CI**	***I*^2^ (%)**	**Overall effect *P*–value**	**Subgroup differences *P*–value**
**Total sessions of treatment (Acupuncture** ***vs*****. CG)**						
≥4 weeks	21	−1.11	−1.46 to −0.77	90	< 0.00001	0.12
< 4 weeks	5	−0.69	−1.10 to −0.29	68	0.0008	
**Needle retention time (Acupuncture vs. CG)**						
≥30 min	21	−1.16	−1.48 to −0.83	88	< 0.00001	0.09
< 30 min	2	−0.76	−1.08 to −0.43	0	< 0.00001	
**Acupuncture frequency (Acupuncture vs. CG)**						
1 time/day	8	−1.07	−1.61 to −0.53	90	0.00001	0.89
< 1 time/day	14	−1.12	−1.57 to −0.67	91	< 0.00001	
**Total sessions of treatment (Acupuncture plus CG vs. CG)**						
≥4 weeks	12	−1.15	−1.43 to −0.86	76	< 0.00001	0.36
< 4 weeks	3	−1.79	−3.14 to −0.43	95	0.01	
**Needle retention time (Acupuncture plus CG** ***vs*****. CG)**						
≥30 min	12	−1.37	−1.74 to −1.00	85	< 0.00001	0.04^*^
< 30 min	3	−0.81	−1.20 to −0.41	55	< 0.0001	
**Acupuncture frequency (Acupuncture plus CG** ***vs*****. CG)**						
1 time/day	9	−1.41	−1.88 to −0.93	88	< 0.00001	0.27
< 1 time/day	6	−1.06	−1.44 to −0.67	74	< 0.00001	

CG, control group; CI, confidence interval; PSQI, Pittsburgh Sleep Quality Index; SMD, standardized mean difference.

^*^P < 0.05 indicates a significant difference.

### 3.8. Meta-regression analysis

We performed a meta-regression using sample size, mean age, and year of publication as co-variables to query the sources of heterogeneity. The results indicated that there was no linear relationship between the characteristics and the PSQI total score, and these characteristics were not the source of heterogeneity (Table 3).

**Table 3:** Meta-regression of the total PSQI score for the two interventions.

**Characteristic**	**Regression coefficient**	**Standard error**	***t***	***P* > |t|**	**95% CI**
**Acupuncture vs. CG**					
Year of publication	−0.0005	0.010	−0.05	0.960	−0.022 to 0.021
Sample size	−0.001	0.002	−0.40	0.689	−0.006 to 0.004
Mean age	−0.003	0.011	−0.30	0.769	−0.026 to 0.020
**Acupuncture plus CG** ***vs*****. CG**					
Year of publication	0.008	0.034	0.25	0.808	−0.065 to 0.083
Sample size	−0.001	0.002	−0.57	0.576	−0.005 to 0.003
Mean age	−0.001	0.014	−0.07	0.943	−0.032 to 0.030

CG, control group; PSQI, Pittsburgh Sleep Quality Index.

### 3.9. Risk of publication bias

We performed a publication bias analysis of the PSQI scores for the two interventions. Funnel plots were asymmetric (Figures 3C, 4C) and Egger's regression test (*P*_*Acupuncture*_ _vs._ _*CG*_ < 0.0001, *P*_*Acupuncture plus CG*_ _vs._ _*CG*_ < 0.0001) showed a publication bias (Supplementary Figures S3, S4). Therefore, we conducted trim-and-fill test analysis to assess the effect of publication bias on the interpretation of the results, and the result indicated that this publication bias did not affect the estimates, although several RCTs showing negative findings remained unpublished (Figures 3D, 4D and Table 4).

**Table 4:** Trim-and-fill test of the total PSQI scores for the two interventions.

**Outcome**	**Effect-size**	**Effect model**	**Before trim-and-fill**		**After trim-and-fill**		**Increased research**
			**Pooled estimate**	**95%CI**	**Pooled estimate**	**95%CI**	
Acupuncture vs. CG	SMD	FE	−0.83	−0.92 to −0.73	−1.148	−1.23 to −1.06	6
		RE	−1.04	−1.33 to −0.74	−1.299	−1.62 to −0.97	
Acupuncture plus CG vs. CG	SMD	FE	−1.16	−1.28 to −1.04	−1.236	−1.35 to −1.11	1
		RE	−1.26	−1.57 to −0.94	−1.338	−1.66 to −1.01	

CG, control group; FE, fixed-effects; PSQI, Pittsburgh Sleep Quality Index; RE, random-effects; SMD, standardized mean difference.

### 3.10. Secondary outcomes

#### 3.10.1. Long-term efficacy

**Figure 6:**
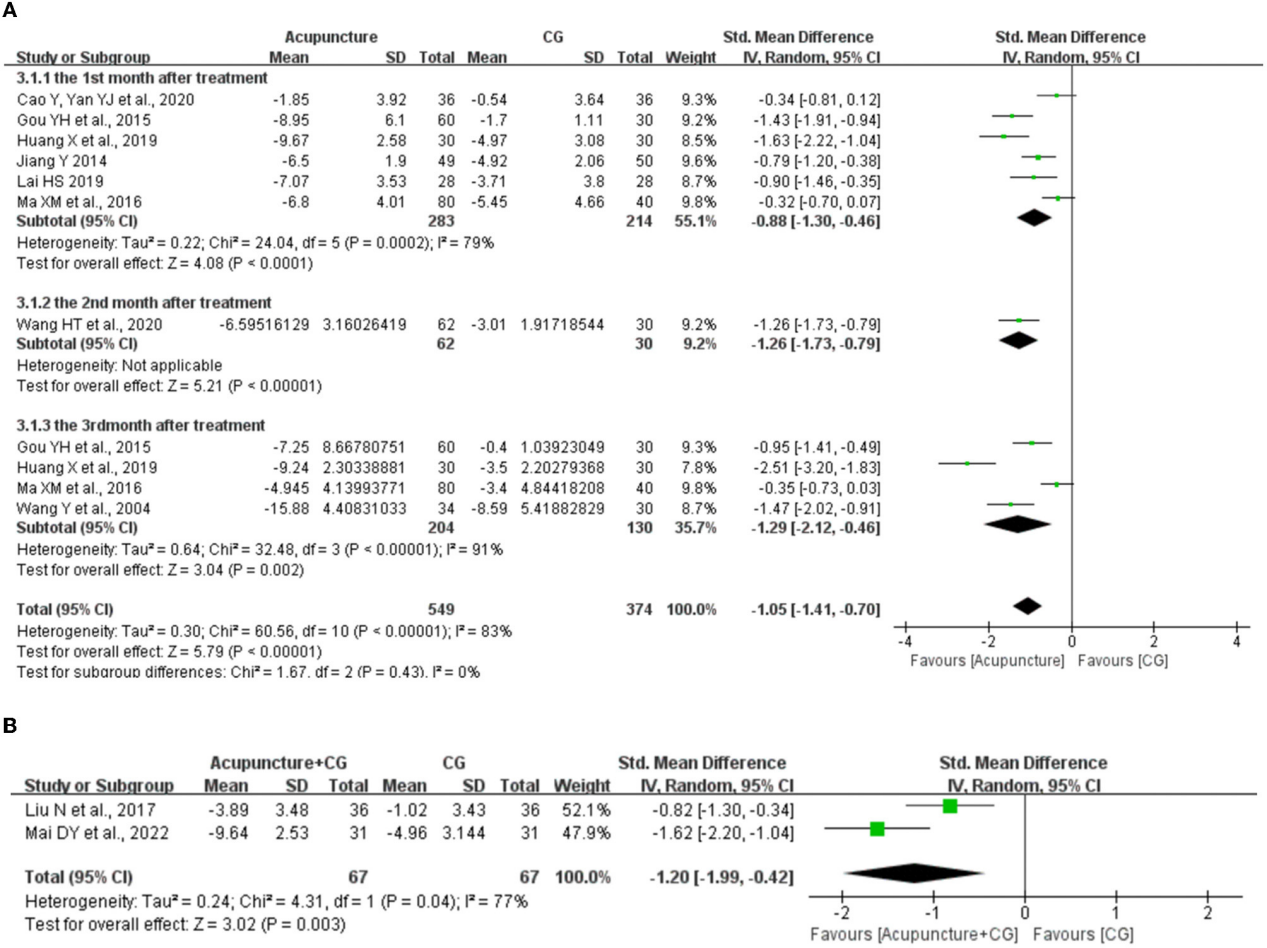
The forest plot of acupuncture for long-term efficacy. **(A)** The long-term efficacy of acupuncture. **(B)** The long-term efficacy of acupuncture plus control group (CG). CI, confidence interval; SD, standard deviation.

According to the follow-up visits at the 1st month, 2nd month, and 3rd month after the treatment, we analyzed the long-term efficacy of acupuncture. Eight studies (38, 43, 47, 49, 54, 56, 59, 68) evaluated the long-term efficacy for acupuncture compared with CG, the random-effects meta-analysis showed that acupuncture had long-term efficacy at the 1st month (*SMD* = −0.88, 95% CI: −1.30 to −0.46, *P* < 0.0001) and 3rd month (*SMD* = −1.29, 95% CI: −2.12 to −0.46, *P* = 0.0002) in ameliorating sleep quality. Moreover, one study (43) reported that acupuncture had long-term efficacy (2nd month) in ameliorating sleep quality (Figure 6A).

We also evaluated the long-term efficacy for acupuncture plus CG compared with CG, the random-effects meta-analysis showed that acupuncture plus CG had long-term efficacy at the 1st month (*SMD* = −1.20, 95% CI: −1.99 to −0.42, *P* = 0.04) in ameliorating sleep quality compared with CG, and one study (28) reported that acupuncture plus CG had long-term efficacy (3rd month) in ameliorating sleep quality (Figure 6B).

### 3.11. Objective sleep data

**Figure 7:**
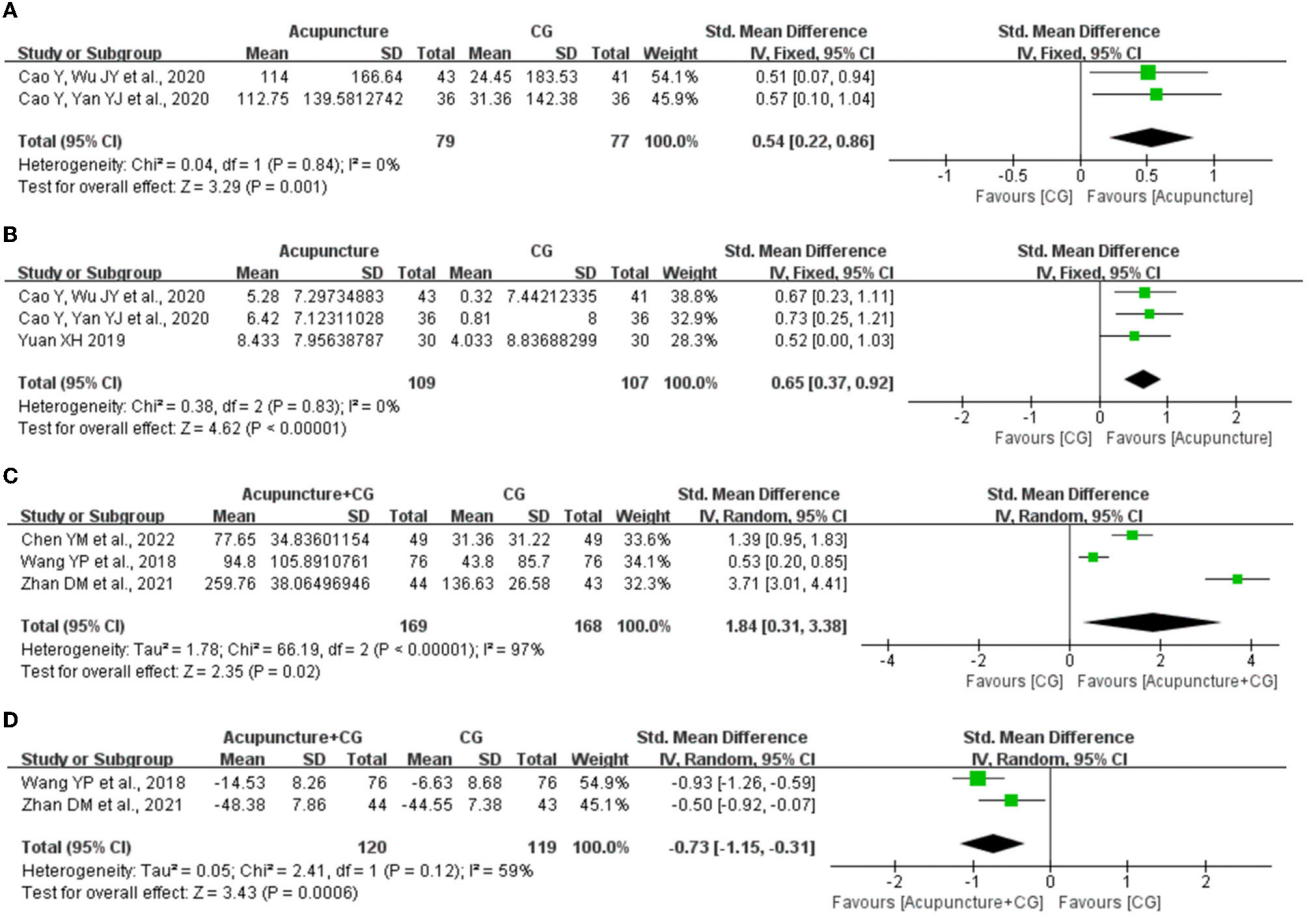
The forest plot of acupuncture for the improvement of objective sleep data. **(A)** Effect of acupuncture on total sleep time. **(B)** Effect of acupuncture sleep efficiency. **(C)** Effect of acupuncture plus control group (CG) on total sleep time. **(D)** Effect of acupuncture plus CG on sleep latency. CI, confidence interval; SD, standard deviation.

Objective sleep data collected by PSG was also used to evaluate the effect of acupuncture on improving sleep quality. Three studies (38, 45, 48) evaluated the objective sleep data for acupuncture compared with CG, and the fixed-effects meta-analysis showed superior effects of acupuncture on total sleep time (*SMD* = 0.54, 95% CI: 0.22 to 0.86, *P* = 0.001, Figure 7A) and sleep latency (*SMD* = 0.65, 95% CI: 0.37 to 0.92, *P* < 0.00001, Figure 7B). Furthermore, three studies (29, 31, 52) evaluated the objective sleep data for acupuncture plus CG compared with CG, and the random-effects meta-analysis showed superior effects of acupuncture plus CG on total sleep time (*SMD* = 1.84, 95% CI: 0.31 to 3.38, *P* = 0.02, Figure 7C) and sleep latency (*SMD* = −0.73, 95% CI: −1.15 to −0.31, *P* = 0.0006, Figure 7D).

### 3.12. Adverse events

Only a total of 6 (36, 38, 45, 54, 58, 64) out of the included 41 studies reported on safety measures. Out of these two studies (58, 64) reported that no adverse events associated with acupuncture treatment occurred, and four (36, 38, 45, 54) reported acupuncture-related adverse events which were mild and did not require treatment, including bleeding at the needle site, perspiration, dizziness, and nausea. However, as only about 15% of the included information about the safety of the investigated interventions no reliable conclusions on safety can be draw.

### 3.13. Quality of evidence

We used GRADE to assess the quality of evidence. The results showed Low or very low level of evidence concerned with the fact that acupuncture can improve PSQI, increase sleep efficiency and total sleep time, reduce sleep latency, and has a long-term efficacy because of the methodological limitations, small size of the optimal information sample and the risk of publication bias (Table 5).

**Table 5:** Quality of evidence for the included studies.

**No**.	**Study design**	**Certainty assessment**					**Summary of results**						**Importance**
		**Risk of bias**	**Inconsistency**	**Indirectness**	**Imprecision**	**Other considerations**	**No of patients**		**Effect (95%CI)**		**Certainty**		
							**T**	**C**	**Relative**	**Absolute**			
**Acupuncture vs. CG on PSQI**												
26	RCT	Serious ^**a**^	Serious ^**b**^	not Serious	not Serious	Serious ^**d**^	1,083	889	-	SMD 1.03 lower (1.32 lower to 0.74 lower)	⊕○○○	Very low	Critical
**Acupuncture plus CG vs. CG on PSQI**												
15	RCT	Serious ^**a**^	Serious ^**b**^	not Serious	not Serious	Serious ^**d**^	629	602	-	SMD 1.26 lower (1.57 lower to 0.94 lower)	⊕○○○	Very low	Critical
**Acupuncture vs. CG on long-term efficacy (1st month after the treatment)**												
6	RCT	Serious ^**a**^	Serious ^**b**^	not serious	not Serious	Serious	283	214	-	SMD 0.88 lower (1.30 lower to 0.46 lower)	⊕⊕○○	low	Important
**Acupuncture vs. CG on long-term efficacy (3rd month after the treatment)**												
4	RCT	Serious ^**a**^	Serious ^**b**^	not serious	not Serious	not Serious	204	130		SMD 1.29 lower (2.12 lower to 0.46 lower)	⊕○○○	Very low	Important
**Acupuncture plus CG vs. CG on long-term efficacy (1st month after the treatment)**												
2	RCT	not Serious	Serious ^**b**^	not serious	Serious ^**c**^	Serious ^**e**^	67	67	-	SMD = 1.20 lower (1.99 lower to 0.42 lower)	⊕○○○	Very low	Important
**Acupuncture vs. CG on total sleep time**												
2	RCT	not serious	not serious	not serious	Serious ^**c**^	Serious ^**e**^	79	77	-	SMD = 0.54 higher (0.22 higher to 0.86 higher)	⊕⊕○○	Low	Important
**Acupuncture vs. CG on sleep efficiency**												
3	RCT	not Serious	not Serious	not serious	Serious ^**c**^	Serious ^**e**^	109	107	-	SMD 0.65 higher (0.37 higher to 0.92 higher)	⊕⊕○○	Low	Important
3	RCT	not Serious	Serious ^**b**^	not serious	not serious	Serious ^**e**^	169	168		SMD 1.84 higher (0.31 higher to 3.38 higher)	⊕⊕○○	Low	Important
**Acupuncture plus CG vs. CG on sleep latency**												
2	RCT	not Serious	Serious ^**b**^	not serious	Serious ^**c**^	Serious ^**e**^	120	119	-	SMD = 0.73 lower (1.15 lower to 0.31 lower)	⊕○○○	Very low	Important

RCT, randomized controlled trial; SMD, standardized mean difference; CI, confidence interval.

^a^Downgrade by one level: The adequate randomization process was described in all studies, and several studies used allocation concealment and blinded design, while other risks of bias domains were also acceptable. Therefore, the included studies were considered to have mild methodological limitations.

^b^Downgrade by one level: heterogeneity across the studies was fairly high, but the overlapping degree of confidence intervals was acceptable.

^c^Downgrade by one level: The optimal information sample size was small.

^d^Downgrade by one level: There was a risk of publication bias.

^e^Downgrade by one level: The number of RCTs was very small.

## 4. Discussion

This study reviewed the efficacy, long-term effects, and safety of acupuncture for post-stroke insomnia. A total of 41 studies were included in the quantitative synthesis. Pooled results indicated that acupuncture or acupuncture plus CG was superior to CG in improving sleep quality and long-term effects. In terms of safety, acupuncture did not cause serious adverse reactions, however, due to limited safety information, reliable safety conclusions cannot be drawn. In addition, the results of the objective sleep data showed that compared with the control groups, acupuncture increased sleep efficiency and total sleep time, and acupuncture plus CG reduced sleep latency and increased total sleep time. Acupuncture therapy is an important component of traditional Chinese medicine and has been recommended within several clinical practice guidelines all over the world (69). Studies have shown acupuncture to be effective in the treatment of stable angina (70), stress urinary incontinence (71) and neurological disorders (72). Current research study (73) found that acupuncture can regulate the post-receptor signaling pathways of 5-HT1A and 5-HT2A and improve the mechanism of central 5-HT disorder, thus exerting sleep-promoting effects. Furthermore, animal study (74) has shown that acupuncture can regulate GABA-Gln metabolism, increase GABA levels, and upregulate the expression of GABAA receptors to mediate central nervous system function in rats. Another study (75) has shown that acupuncture can regulate MT content and MT1 and MT2 mRNA expression in the ventral lateral preoptic area of the hypothalamus of insomniac rats, thus improving insomnia. In addition, acupuncture can improve insomnia by increasing parasympathetic activity and inhibiting sympathetic activity, oxidative stress, and inflammation (76, 77).

At the same time, we assessed the impact of different treatment strategies, sessions of acupuncture, acupuncture frequencies, and needle retention times on the therapeutic effect, which can be clinically significant. Through the subgroup analysis, we found superior effects of acupuncture compared to BZDs, non-BZDs, or other treatments, regardless of whether acupuncture alone or acupuncture plus CG. Moreover, post-infarction patients were the most represented subgroup of participants in the included studies, we found positive effects of acupuncture regardless of whether acupuncture compared to sham acupuncture or acupuncture plus CG compared to CG; however, the efficacy of acupuncture alone compared to non-BZDs or other hypnotics needs further research. Follow-up data can reflect the long-term clinical effect, and we found that acupuncture treatment had long-term effect on improving PSQI scores in post-stroke insomnia at the 1st, 2nd, and 3rd month after the treatment. As for the total sessions of treatment, acupuncture frequency, and needle retention time, we found that acupuncture could lower the PSQI score regardless of whether the total sessions of treatment lasted < 4 weeks or ≥4 weeks, the acupuncture frequency was < 1 time/day or 1 time/day, and the needle retention time lasted < 30 min or ≥30 min. Furthermore, we found that once a day acupuncture treatment or a greater time of needle retention might be associated with better sleep quality effects. We also assessed the acupuncture treatment regimen using STRICTA and calculated frequency statistics on the acupuncture points. Most of the included studies failed to adequately report acupuncture details according to STRICTA criteria. A total of 52 acupuncture points with frequencies ranging from 1 to 26 were used; the commonly used acupuncture points for post-stroke insomnia were GV20, HT7, SP6, GV24, EX-HN1, PC6, Anshen point, and GV29.

However, given the high risk of bias, the level of evidence was low or very low. The included studies had a risk of bias in terms of quality assessment, and most studies had at least one domain rated as unclear risk of bias in the six dimensions assessed. Most of the studies failed to perform assignment concealment. Studies with inadequate blinding or allocation concealment may be subject to the potential risk of bias and exaggerated treatment effects. The trim-and-fill test indicated that publication bias did not affect the estimates, although the results of the funnel plot and Egger's regression test indicated potential publication bias. Moreover, heterogeneity was evaluated in this study. We found no significant sources of heterogeneity by performing meta-regression and subgroup analysis, and sensitivity analysis showed that the results were robustness. Despite these limitations and the low level of evidence, this meta-analysis provides valuable information regarding acupuncture in the post-stroke insomnia population. It also provides information on the long-term efficacy of acupuncture and its effects on special populations such as those with post-infarction insomnia.

Similar to the results of this study, previous SRs showed that acupuncture is effective and safe in treating post-stroke insomnia. However, this study has some advantages over previous SRs. First, our study included recently published trials. Second, objective sleep indicators such as PSG results were used to evaluate the effect of acupuncture, which increased the credibility of the evidence. Third, follow-up data were included as outcome indicators to demonstrate the long-term clinical efficacy of acupuncture for post-stroke insomnia. Fourth, this study used sensitivity analysis, subgroup analysis, and meta-regression to explore the causes of heterogeneity, the stability of the meta-analysis results, and the influence of some characteristics on the efficacy of acupuncture treatment. Fifth, we used funnel plots and Egger's regression tests to assess the possibility of publication bias and used trim-and-fill analysis to assess the effect of publication bias on the interpretation of results. Finally, the current study successfully avoided the flaws of previous SRs and updated the evidence regarding the effects of acupuncture on post-stroke insomnia. However, This study has several limitations. First, most of the included studies had a risk of bias in terms of quality assessment, which weakened the credibility of the evidence. Second, different studies used different diagnostic criteria for insomnia, such as the DSM-5, CCMD-3, and ICD-10, which may have affected the evaluation of the acupuncture effect. Third, all included studies were carried out in China, which might potentially contribute to bias. Therefore, the conclusions drawn need to be interpreted with caution. Finally, the studies were heterogeneous with respect to interventions, such as the selection of acupuncture points of the included studies, and most of the included studies failed to adequately report acupuncture details according to STRICTA criteria.

This study has some interesting implications for future research. First, we found that most control treatments in studies were BZDs, while higher annual doses or long-term use of BZDs can increase stroke incidence (12, 13). We suggest using acupuncture or acupuncture combined with short-term, intermittent, or low-dose sleeping pills for the treatment of post-stroke insomnia in the future; however, more studies are needed to identify the guidelines for sleeping pills (such as dose, course of treatment, and frequency) when in combination with acupuncture. Second, acupuncture treatment should be regulated according to STRICTA, and the frequency, course of treatment, and needle retention time of acupuncture should be investigated further in future studies. Third, acupuncture can improve sleep quality and has long-term efficiency; moreover, it can also relieve other stroke sequelae such as depression (17) to avoid aggravating insomnia. These benefits can be used to prevent post-stroke insomnia from becoming chronic. Finally, the heterogeneity of the meta-analysis results of objective outcomes was low, while that of the subjective outcome was high. Potential clinical heterogeneity may have been caused by the process of subjective outcome collection. In the future, we should standardize the selection of research indicators as well as the operation of subjective indicators and increase the number of objective indicators.

## 5. Conclusion

This study suggested that acupuncture has positive effects on subjective sleep quality, long-term clinical efficacy in post-stroke insomnia. Moreover, the results of objective sleep indicators showed that acupuncture can improve sleep efficiency and total sleep time and reduce sleep latency. However, due to limited safety information, reliable safety conclusions cannot be drawn. Furthermore, owing to the risk of bias and heterogeneity of the included studies, the level of evidence was low, and more studies with rigorous designs and larger sample sizes are needed to validate our results.

## Data availability statement

The original contributions presented in the study are included in the article/Supplementary material, further inquiries can be directed to the corresponding authors.

## Author contributions

MS, MZ, and JZ conceived and drafted the study. MS and TS carried out the literature searches and extracted the data. MS, HH, and ZC performed the statistical analysis. MS and ZC contributed to manuscript drafting. JZ and FY oversaw the conduct of the study. All authors have read, critically reviewed, and approved the final manuscript.
