# Supplementary material for: Efficacy and safety of acupuncture on sleep quality for post-stroke insomnia: a systematic review and meta-analysis
Source: Front Neurol. 2023 Jun 27;14:1164604. doi: 10.3389/fneur.2023.1164604 (PMC10333537; doi:10.3389/fneur.2023.1164604)
Supplement: Supplementary file 1 [file Data_Sheet_1.docx]

# Supplementary Material

# Acupuncture for post-stroke insomnia: A systematic review and meta-analysis

Menglong Shi^1^, Zhaochen Ji^1^, Tianye Sun^2^, Haiyin Hu^1^, Zhe Chen^1^, Chaonan Feng^1^, Junhua Zhang^1^, Min Zhao^3 *^, Fengwen Yang^1*^

^1^Evidence-Based Medicine Center, Tianjin University of Traditional Chinese Medicine, Tianjin, China

^2^Dongfang Hospital, Beijing University of Chinese Medicine, Beijing, China

^3^The First Affiliated Hospital of Henan University of CM, Zhengzhou, China

**Table S1: Search strategies of PubMed (Searched from inception to Sept. 12, 2022 and found 34 literature)**

| **#** | **Searches** |
| --- | --- |
| **#1（Patient）** | ((stroke* [Title/Abstract] OR cerebrovascular disorders*[Title/Abstract] OR brain ischemia*[Title/Abstract] OR cerebral hemorrhage*[Title/Abstract] OR cerebral infarction* [Title/Abstract] OR cerebrovascular accident[Title/Abstract] OR CVA[Title/Abstract] OR brain vascular accident[Title/Abstract] OR apoplexy[Title/Abstract] OR cerebral infarction[Title/Abstract]) OR (stroke [Mesh] OR cerebrovascular disorders [Mesh] OR brain ischemia [Mesh] OR cerebral hemorrhage [Mesh] OR cerebral infarction[Mesh])) |
| **#2（Patient）** | ((("Sleep Initiation and Maintenance Disorders"[Mesh])) OR ((Wakefulness"[Mesh]))) OR ((Sleep Wake Disorders"[Mesh]))) OR (((Early Awakening[Title/Abstract]) OR (Awakening, Early[Title/Abstract]) OR (Nonorganic Insomnia[Title/Abstract]) OR (Insomnia, Nonorganic[Title/Abstract]) OR (Primary Insomnia[Title/Abstract]) OR (Insomnia, Primary[Title/Abstract]) OR (insomn*[Title/Abstract]) ) OR (（DIMS (Disorders of Initiating and Maintaining Sleep））[Title/Abstract])))) |
| **#3（Intervention）** | (((acupuncture therapy [Mesh] OR acupuncture [Mesh] OR electroacupuncture [Mesh]) OR (acupuncture*[Title/Abstract] OR needling [Title/Abstract] OR electroacupuncture* [Title/Abstract] OR electro-acupuncture [Title/Abstract] OR moxibustion [Title/Abstract] OR acupoint [Title/Abstract])) |
| **#4（Study Design）** | (((((((randomized controlled trial[Publication Type]) OR (controlled clinical trial[Publication Type])) OR (Clinical Trials[Publication Type])) OR (placebo[Publication Type])) OR (random*[Publication Type])) OR (allocate*[Publication Type])) OR (assign*[Publication Type])) OR (RCT*[Publication Type]) |
| **#5** | **#1 AND #2 AND #3 AND #4** |

**Table S2:** Sear**ch strategies of EMbase (Searched from inception to Sept. 12, 2022 and found 49 literature)**

| **#** | **Searches** |
| --- | --- |
| **#1（Patient）** | 'cerebrovascular accident'/exp OR 'stroke*':ti,ab OR 'cerebrovascular disorders*':ti,ab OR 'brain ischemia*':ti,ab OR 'cerebral hemorrhage*':ti,ab OR 'cerebral infarction*':ti,ab OR 'cerebrovascular accident':ti,ab OR 'CVA':ti,ab OR 'brain vascular accident':ti,ab OR 'apoplexy':ti,ab OR 'cerebral infarction' |
| **#2（Patient）** | 'insomnia'/exp OR 'Early Awakening':ti,ab OR 'Awakening, Early':ti,ab OR 'Early Awakening':ti,ab OR 'Nonorganic Insomnia':ti,ab OR 'Insomnia, Nonorganic':ti,ab OR 'Primary Insomnia':ti,ab OR 'Insomnia, Primary':ti,ab OR 'insomn*':ti,ab OR 'DIMS (Disorders of Initiating and Maintaining Sleep)':ti,ab |
| **#3（Intervention）** | 'acupuncture'/exp OR 'acupuncture*':ti,ab OR 'needling':ti,ab OR 'electroacupuncture*':ti,ab OR 'electro-acupuncture':ti,ab OR 'moxibustion':ti,ab OR 'acupoint':ti,ab |
| **#4（Study Design）** | 'randomized controlled trial':ti,ab OR 'controlled clinical trial':ti,ab OR 'randomized':ti,ab OR 'placebo':ti,ab OR 'random*':ti,ab OR 'allocate*':ti,ab OR 'assign*':ti,ab OR 'RCT*':ti,ab |
| **#5** | **#1 AND #2 AND #3 AND #4** |

**Table S3: Search strategies of Web of Science (Searched from inception to Sept. 12, 2022 and found 11 literature)**

| **#** | **Searches** |
| --- | --- |
| **#1（Patient）** | stroke* (Topic) or cerebrovascular disorders* (Topic) or brain ischemia* (Topic) or cerebral hemorrhage* (Topic) or cerebral infarction* (Topic) or cerebrovascular accident (Topic) or CVA (Topic) or brain vascular accident (Topic) or Apoplexy (Topic) or cerebral infarction (Topic) |
| **#2（Patient）** | Sleep Initiation and Maintenance Disorders (Topic) or Wakefulness (Topic) or Sleep Wake Disorders (Topic) or Early Awakening (Topic) or Awakening, Early (Topic) or Nonorganic Insomnia (Topic) or Insomnia, Nonorganic (Topic) or Primary Insomnia (Topic) or insomn* (Topic) or Insomnia, Primary (Topic) or DIMS (Disorders of Initiating and Maintaining Sleep) (Topic) |
| **#3（Intervention）** | acupuncture therapy (Topic) or Acupuncture (Topic) or Electroacupuncture (Topic) or acupuncture* (Topic) or Needling (Topic) or electroacupuncture* (Topic) or electro-acupuncture (Topic) or Moxibustion (Topic) or acupoint (Topic) |
| **#4（Study Design）** | randomized controlled trial (Topic) OR controlled clinical trial (Topic) or Clinical Trials (Topic) or placebo (Topic) or random* (Topic) or allocate* (Topic) or assign* (Topic) or RCT* (Topic) |
| **#5** | **#1 AND #2 AND #3 AND #4** |

**Table S4: Search strategies of Cochrane Central Register of Controlled Trials (CENTRAL) (Searched from inception to Sept. 12, 2022 and found 37 literature)**

| **#** | **Searches** |
| --- | --- |
| **#1（Patient）** | MeSH descriptor: [Stroke] explode all trees  MeSH descriptor: [Cerebrovascular Disorders] explode all trees  MeSH descriptor: [Cerebral Hemorrhage] explode all trees  (stroke*):ti,ab,kw OR (cerebrovascular disorders*):ti,ab,kw OR (brain ischemia*):ti,ab,kw OR (cerebral hemorrhage*):ti,ab,kw OR (cerebral infarction*):ti,ab,kw  (cerebrovascular accident):ti,ab,kw OR (CVA):ti,ab,kw OR (brain vascular accident):ti,ab,kw OR (Apoplexy):ti,ab,kw OR (cerebral infarction):ti,ab,kw |
| **#2（Patient）** | MeSH descriptor: [Sleep Initiation and Maintenance Disorders] explode all trees  (Wakefulness):ti,ab,kw OR (Sleep Wake Disorders):ti,ab,kw OR (Early Awakening):ti,ab,kw OR (Awakening, Early):ti,ab,kw OR (Nonorganic Insomnia):ti,ab,kw  (Insomnia, Nonorganic):ti,ab,kw OR (insomn*):ti,ab,kw OR (Insomnia, Primary):ti,ab,kw OR (Primary Insomnia):ti,ab,kw |
| **#3（Intervention）** | **MeSH descriptor: [Acupuncture] explode all trees**  **MeSH descriptor: [Acupuncture Therapy] explode all trees**  **(Moxibustion):ti,ab,kw OR (acupuncture*):ti,ab,kw OR (Needling):ti,ab,kw OR (electroacupuncture*):ti,ab,kw OR (acupoint):ti,ab,kw** |
| **#4（Study Design）** | **(**randomized controlled trial**):ab,ti,pt OR (**controlled clinical trial**):ab,ti,pt OR (**Clinical Trials**):ab,ti,pt OR (**placebo**):ab,ti,pt OR (**random***):ab,ti,pt OR (**allocate***):ab,ti,pt OR (**assign***):ab,ti,pt OR (**RCT***):ab,ti,pt** |
| **#5** | **#1 AND #2 AND #3 AND #4** |

**Table S5: Search strategies of CNKI (Searched from inception to Sept. 12, 2022 and found 359 literature)**

| **#** | **Searches** |
| --- | --- |
| **#1（Patient）** | SU=('失眠'+'不寐'+'失眠障碍') |
| **#2（Patient）** | SU=('卒中'+'中风'+'脑出血'+'脑梗死'+'脑血管意外'+'脑梗'+'脑缺血'+'脑梗塞'+'脑血栓'+'脑栓塞'+'缺血性脑卒中'+'缺血性中风') |
| **#3（Intervention）** | SU=('针'+'刺'+'灸'+'针刺'+'电针'+'火针'+'温针'+'毫针'+'针灸'+'温针灸'+'艾灸'+'头针') |
| **#4（Study Design）** | SU=('随机'+'对照'+'盲法'+'临床试验'+'治疗'+'分组'+'安慰剂') |
| **#5** | **#1 AND #2 AND #3 AND #4** |

**Table S6: Search strategies of Wanfang (Searched from inception to Sept. 12, 2022 and found 710 literature)**

| **#** | **Searches** |
| --- | --- |
| **#1（Patient）** | ("失眠" or "不寐" or "失眠障碍") |
| **#2（Patient）** | ("卒中" or "中风" or "脑出血" or "脑梗死" or "脑血管意外" or "脑梗" or "脑缺血" or "脑梗塞" or "脑血栓" or "脑栓塞" or "缺血性脑卒中" or "缺血性中风") |
| **#3（Intervention）** | ("针" or "刺" or "灸" or "针刺" or "电针" or "火针" or "温针" or "毫针" or "针灸" or "温针灸" or "艾灸" or "头针") |
| **#4（Study Design）** | ("随机" or "对照" or "盲法" or "临床试验" or "治疗" or "分组" or "安慰剂") |
| **#5** | **#1 AND #2 AND #3 AND #4** |

**Table S7: Search strategies of VIP (Searched from inception to Sept. 12, 2022 and found 428 literature)**

| **#** | **Searches** |
| --- | --- |
| **#1（Patient）** | U=("失眠" OR "不寐" OR "失眠障碍") |
| **#2（Patient）** | U=("卒中" OR "中风" OR "脑出血" OR "脑梗死" OR "脑血管意外" OR "脑梗" OR "脑缺血" OR "脑梗塞" OR "脑血栓" OR "脑栓塞" OR "缺血性脑卒中" OR "缺血性中风" |
| **#3（Intervention）** | U=("针" OR "刺" OR "灸" OR "针刺" OR "电针" OR "火针" OR "温针" OR "毫针" OR "针灸" OR "温针灸" OR "艾灸" OR "头针") |
| **#4（Study Design）** | U=("随机" OR "对照" OR "盲法" OR "临床试验" OR "治疗" OR "分组" OR "安慰剂") |
| **#5** | **#1 AND #2 AND #3 AND #4** |

**Table S8: Search strategies of SinoMed (Searched from inception to Sept. 12, 2022 and found 590 literature)**

| **#** | **Searches** |
| --- | --- |
| **#1（Patient）** | ("失眠" or "不寐" or "失眠障碍") |
| **#2（Patient）** | ("卒中" or "中风" or "脑出血" or "脑梗死" or "脑血管意外" or "脑梗" or "脑缺血" or "脑梗塞" or "脑血栓" or "脑栓塞" or "缺血性脑卒中" or "缺血性中风") |
| **#3（Intervention）** | ("针" or "刺" or "灸" or "针刺" or "电针" or "火针" or "温针" or "毫针" or "针灸" or "温针灸" or "艾灸" or "头针") |
| **#4（Study Design）** | ("随机" or "对照" or "盲法" or "临床试验" or "治疗" or "分组" or "安慰剂") |
| **#5** | **#1 AND #2 AND #3 AND #4** |

**Table S9. The excluded 18 studies and reasons**

| **Excluded studies** | **Reasons** |
| --- | --- |
| Qin et al., 2019 (Qin et al., 2019) | No outcome of interest |
| Wang et al., 2010 (Wang et al., 2016) | No outcome of interest |
| Tan et al., 2014 (Tan, 2019) | No outcome of interest |
| Jin et al., 2014 (Jin et al., 2021) | No outcome of interest |
| Jiao et al., 2014 (Jiao et al., 2021) | No outcome of interest |
| Zhang et al., 2014 (Zhang et al., 2020) | No outcome of interest |
| Ye et al., 2014 (Ye et al., 2010) | No outcome of interest |
| Wang et al., 2014 (Wang and Zhao, 2012) | No outcome of interest |
| Huang et al., 2014 (Huang, 2012) | No outcome of interest |
| Jin et al., 2014 (Jin, 2016) | No outcome of interest |
| Wu et al., 2014 (Wu et al., 2013) | Not the target intervention |
| Huang et al., 2014 (Huang and Wang, 2011) | Not the target intervention |
| Jiang et al., 2014 (Jiang, 2018) | Not the target intervention |
| Li et al., 2014 (Li, 2021) | Not the target intervention |
| Huan et al., 2014 (Huan, 2020) | Not the target intervention |
| Ran et al., 2014 (Ran, 2013) | Not the target intervention |
| Hong et al., 2014 (Hong, 2021) | Not the target patients |
| Li et al., 2014 (Li et al., 2020) | Not the study design |

**References:**

Huan, H., 2020. Clinical reasearch on the effect of stroke-promoting moxibustion on sleep disorder after ischemic stroke., Nanjing University Of Chinese Medicine, p. 57.

Hong, B.Q., 2021. Clinical observation of regulating spirit acupuncture technique combined with drugs in treatment of post-stroke circadiam sleep-wake disorder., Guangzhou University of Chinese Medicine, p. 63.

Huang, J.M., Wang, K.H., 2011, Treatment of 30 Cases of Insomnia after Stroke with Acupuncture and Medicine. SHAANXI JOURNAL OF TRADITIONAL CHINESE MEDICINE 32, 1227-1228.

Huang, M., 2012, Observation on the effect of acupuncture and moxibustion on patients with sleep disorder after stroke. Journal Of Clinical Acupuncture And Moxibustion 28, 15-16.

Jiang, Y.Y., 2018. Effect of electroacupuncture at EX-HN1 on sleep and cognitive function in patients with insomnia after stroke., Heilongjiang University of Chinese Medicine, p. 55.

Jiao, L.N., Liu, Z.Q., Wang, Y.H., 2021, Treatment of 29 cases of insomnia after stroke with wrist acupuncture. GUANGMING JOURNAL OF CHINESE MEDICINE 36, 1302-1304.

Jin, X., Wang, N.N., Zou, Y., Gao, D., Liu, J., 2021, Clinical observation on Relieving insomnia of Apoplexy Patients with Psetle and acupuncture. JOURNAL OF LIAONING UNIVERSITY OF TRADITIONAL CHINESE MEDICINE 23, 139-142.

Jin, F.W., 2016, Clinical observation on 30 cases of insomnia caused by phlegm-heat disturbing the heart after stroke treated by acupuncture and moxibustion. World Latest Medicine Information 16, 71-72.

Li, H., 2021, Clinical effect analysis of acupuncture and moxibustion therapy for post-stroke insomnia by invigorating spleen and regulating mind. Health Guide, 118.

Li, M.X., Zhang, Z.Y., Wang, Q., 2020, Efficacy of TongDu TiaoShen Acupuncture on Post-stroke Insomnia and its effects on neurotransmitter level. World Chinese Medicine 15, 112-115.

Qin, H.J., Zhang, Q., Chen, Y.Q., Zhou, S., 2019, The effect of BuShen TongDu acupuncture on sleep quality and quality of life in post-stroke insomnia patients

. Modern Journal of Integrated Traditional Chinese and Western Medicine 28, 3378-3381.

Ran, X., 2013, Clinical efficacy of Integrative Medicine for the treatment of insomnia after stroke. Journal of Aerospace medicine 24, 899-901.

Tan, J.Y., 2019. The clinical study of Jin's Three-needle therapy combined with zolpidem in treating insomnia after stroke., Guangzhou University of Chinese Medicine, p. 42.

Wang, G.M., Zhao, H.S., 2012, Clinical observation of 32 cases of insomnia after stroke treated with acupuncture. Shanxi Journal of Traditional Chinese Medicine 28, 33.

Wang, X.H., Zhou, Z., Wang, Y., 2016, Clinical observation of Tiaoshen- limian acupuncture combined with estazolam on the treatment of insomnia after cerebral infarction. Hebei Journal of Traditional Chinese Medicine 38, 102-105.

Wu, Y.J., Yu, D.D., Chen, H.Y., 2013, Clinical Reasearch of Electric Acupuncture with Auricular Point treatment of Insomnia after stroke. ACTA CHINESE MEDICINE 28, 913-914.

Ye, F.W., Xu, Y.L., Chen, J.W., Xie, Z.M., Wu, Y.Y., 2010, Clinical observation of 30 cases of insomnia after stroke treated with acupuncture at Baihui acupoint. Practical Clinical Journal of Integrated Traditional Chinese and Western Medicine 10, 21-22.

Zhang, Y.Y., Chen, M.Q., Zheng, Z., Fang, P., Zhu, W.J., Chen, Y., 2020, Clinical study on the therapy of warming acupuncture and Moxibustion at Yuan-primary Point in the treatment of Post-stroke insomnia. Chinese Medicine Modern Distance Education of China 18, 88-90.

Table S10. Sequencing of acupuncture points.

| acupuncture point | frequency | acupuncture point | frequency | acupuncture point | frequency | acupuncture point | frequency |
| --- | --- | --- | --- | --- | --- | --- | --- |
| GV20 | 25 | DU11 | 4 | SP10 | 2 | LR14 | 1 |
| HT7 | 24 | KI03 | 4 | Shangen-point | 1 | DU18 | 1 |
| SP6 | 22 | RN12 | 4 | LU5 | 1 | LI11 | 1 |
| GV24 | 19 | DU26 | 3 | SP15 | 1 | DU23 | 1 |
| EX-HN1 | 15 | CV14 | 3 | Ezhongxian | 1 | BL66 | 1 |
| PC6 | 15 | LR03 | 3 | BL13 | 1 | BL40 | 1 |
| Anshen-point | 10 | GB13 | 2 | ST40 | 1 | RN10 | 1 |
| GV29 | 10 | BL18 | 2 | BL17 | 1 | GB14 | 1 |
| ST36 | 7 | LI4 | 2 | SP04 | 1 | GB35 | 1 |
| BL62 | 6 | DU04 | 2 | HT1 | 1 | KL1 | 1 |
| KI06 | 6 | BL23 | 2 | RN14 | 1 | LU10 | 1 |
| RN4 | 5 | EX-HN5 | 2 | PC8 | 1 | DU9 | 1 |
| RN6 | 5 | ST25 | 2 | DU17 | 1 | KI09 | 1 |
| DU16 | 4 | BL15 | 2 | BL20 | 1 |  |  |

Table S11

STRICTA criteria to evaluate the acupuncture treatment protocols

| Author (Year) | needle rationale | Details of needling | | | | | | | Treatment regimen | Complementary interventions | | Practitioner background | Control  intervention |
| --- | --- | --- | --- | --- | --- | --- | --- | --- | --- | --- | --- | --- | --- |
|  |  | No.of needle inserted | Points used | Depth of insertion | Response sought | stimulation mode | Retaining needle time | Needle  type |  | Other interventions | Setting and context of treatment |  |  |
| Mai DY et al, 2022 | Y | N | Ya | N | Y | Y | Y | Y | Y | Y | N | N | Y |
| Chen YM et al., 2022 | Y | N | Ya | N | Y | Y | Y | N | Y | Y | N | N | Y |
| Zhang XL, 2021 | Y | N | Ya | N | N | Y | N | N | Y | Y | N | N | Y |
| Zhan DM et al., 2021 | Y | Y | Ya | Y | Y | Y | Y | Y | Y | Y | N | N | Y |
| Yao XJ et al., 2021 | Y | N | Ya | Y | Y | Y | Y | Y | Y | Y | N | N | Y |
| Li QQ, 2021 | Y | NA | Ya | NA | Y | Y | Y | N | Y | Y | Y | Y | Y |
| Zhang YY et al., 2020 | Y | N | Ya | N | N | Y | Y | N | Y | Y | N | N | Y |
| Wu Z et al., 2020 | Y | Y | Ya | Y | Y | Y | Y | Y | Y | Y | N | N | Y |
| Wang ZH 2020 | Y | N | Ya | Y | Y | Y | Y | N | Y | Y | N | N | Y |
| Sun Y et al., 2020 | Y | Y | Ya | Y | Y | Y | Y | N | Y | Y | N | N | Y |
| Hu YJ et al., 2019 | Y | N | Ya | N | Y | Y | Y | Y | Y | Y | N | N | Y |
| Wang YP et al., 2018 | Y | Y | Ya | N | N | Y | Y | Y | Y | Y | N | N | Y |
| Liu N et al., 2017 | Y | N | Y | Y | N | Y | Y | Y | Y | Y | N | N | Y |
| Han XC 2015 | Y | N | Y | Y | Y | Y | Y | Y | Y | Y | Y | Y | Y |
| Liu JH et al., 2022 | Y | Y | Y | Y | Y | Y | Y | Y | Y | N | N | N | Y |
| Xing Y et al., 2021 | Y | Y | Ya | Y | Y | Y | Y | Y | Y | Y | N | N | Y |
| Zou YL et al., 2020 | Y | Y | Y | N | N | Y | N | Y | Y | N | N | N | Y |
| Wang HT et al., 2020 | Y | Y | Ya | N | Y | Y | Y | N | Y | Y | N | N | Y |
| Sun C 2020 | Y | Y | Ya | Y | Y | Y | N | Y | Y | N | N | N | Y |
| Ye GX et al., 2020 | Y | N | Y | Y | Y | Y | Y | Y | Y | N | N | N | Y |
| He FL et al., 2020) | Y | Y | Y | N | N | Y | Y | N | Y | N | N | N | Y |
| Cao Y, Yan YJ et al., 2020) | Y | Y | Y | Y | N | Y | Y | Y | Y | N | N | N | Y |
| Cao Y, Wu JY et al., 2020 | Y | Y | Y | Y | Y | Y | Y | Y | Y | N | N | N | Y |
| Yuan XH 2019 | Y | Y | Y | Y | Y | Y | Y | Y | Y | N | Y | Y | Y |
| Lai HS 2019 | Y | Y | Y | Y | Y | Y | Y | Y | Y | Y | N | N | Y |
| Huang X et al., 2019 | Y | Y | Y | N | Y | Y | Y | Y | Y | N | N | N | Y |
| Yang K et al., 2019 | Y | N | Ya | N | Y | Y | Y | N | Y | N | N | N | Y |
| Ma XM et al., 2019 | Y | N | Ya | N | N | Y | Y | N | Y | N | N | N | Y |
| Fu L et al., 2016 | Y | Y | Ya | Y | Y | Y | Y | N | Y | N | N | N | Y |
| Tang Y et al., 2015 | Y | N | Ya | Y | N | Y | Y | N | Y | N | N | N | Y |
| Gou YH et al., 2015 | Y | N | Ya | Y | N | Y | N | N | Y | N | N | N | Y |
| Zhu C 2014 | Y | N | Y | N | N | Y | Y | N | Y | N | N | N | Y |
| Jiang Y 2014 | Y | N | Ya | Y | Y | Y | Y | Y | Y | N | N | N | Y |
| Xu YL et al., 2012 | Y | Y | Ya | Y | N | Y | Y | N | Y | N | N | N | Y |
| Chen XJ et al., 2012 | Y | N | Y | N | N | Y | Y | Y | Y | N | N | N | Y |
| Wang Y 2011 | Y | N | Ya | Y | Y | Y | Y | Y | Y | N | N | N | Y |
| Lu YY et al., 2008 | Y | Y | Y | N | N | Y | Y | Y | Y | N | N | N | Y |
| Jia RZ 2010) | Y | N | Ya | N | N | Y | Y | Y | Y | N | N | N | Y |
| Mi JP et al., 2009 | Y | Y | Y | N | Y | Y | Y | N | Y | Y | N | N | Y |
| Li TB 2007 | Y | Y | Ya | Y | N | Y | Y | Y | Y | N | N | N | Y |
| Wang Y et al., 2004 | Y | Y | Ya | Y | Y | Y | Y | Y | Y | N | N | N | Y |

NA, not applicable; NR not reported; Y, reported; Ya, reported but did not mention unilateral or bilateral.


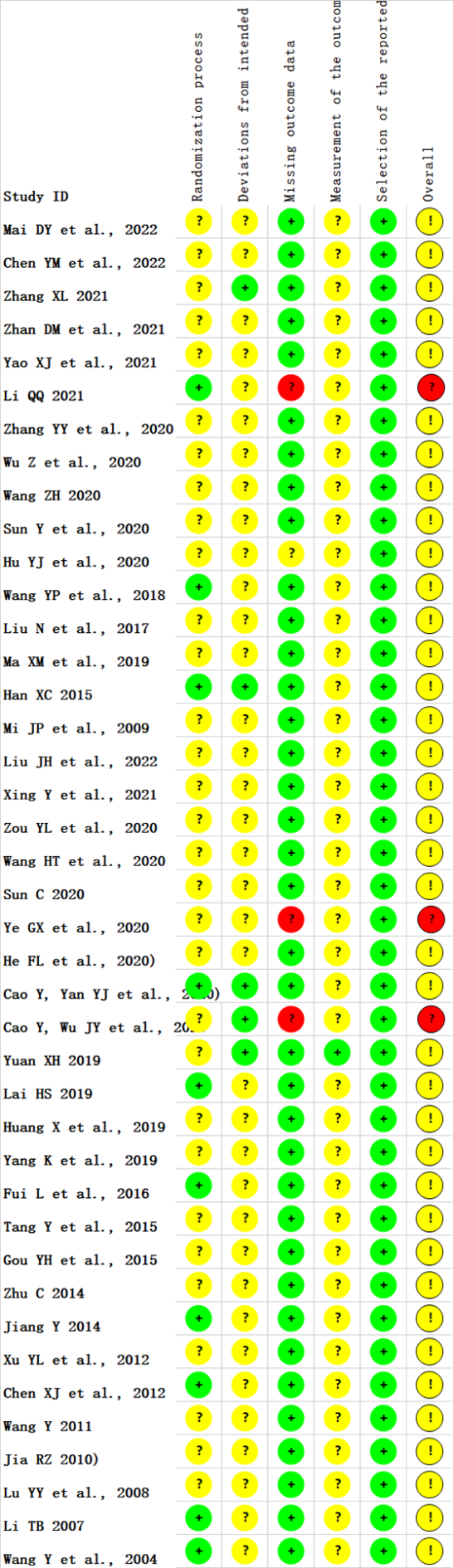


Figure S1. the Risk of bias graph.


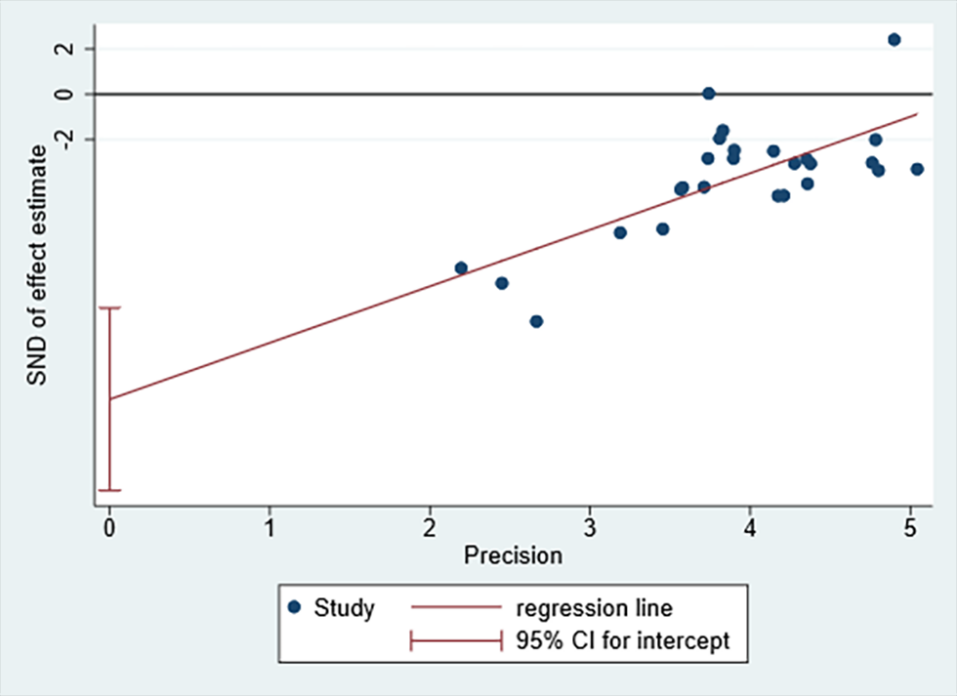


Figure S2 the Egger test of Acupuncture vs CG


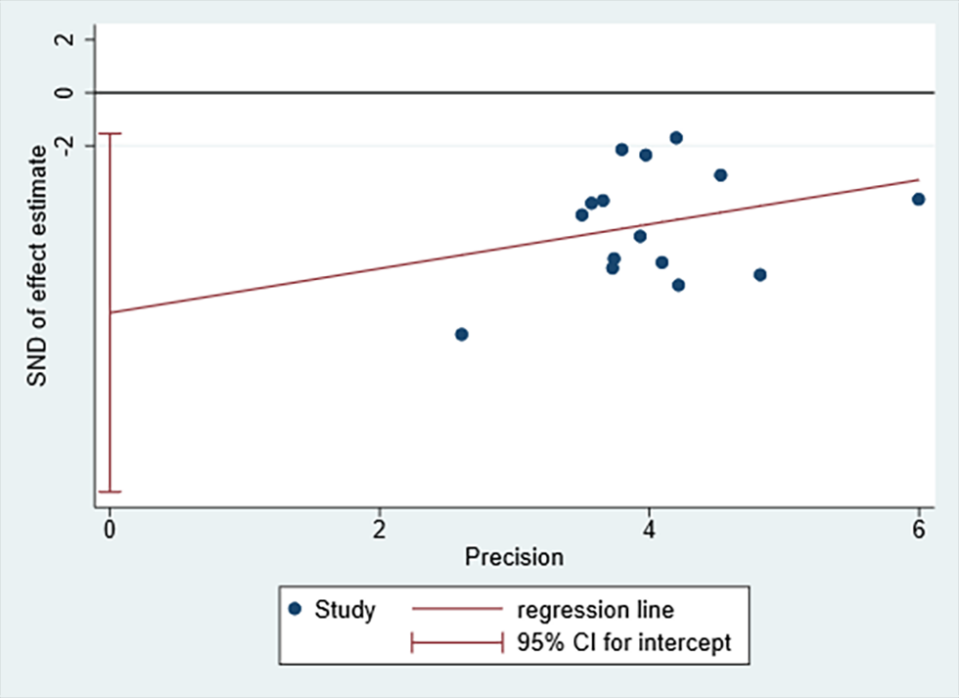


Figure S3 the Egger test of Acupuncture plus CG vs CG
